# Intracochlear Recording of Electrocochleography During and After Cochlear Implant Insertion Dependent on the Location in the Cochlea

**DOI:** 10.1177/23312165241248973

**Published:** 2024-05-08

**Authors:** Sabine Haumann, Max E. Timm, Andreas Büchner, Thomas Lenarz, Rolf B. Salcher

**Affiliations:** 1 Department of Otorhinolaryngology, 9177 Hannover Medical School, Hannover, Germany; 2 Cluster of Excellence “Hearing4All”, Hannover, Germany

**Keywords:** cochlear implant, hearing preservation, CI electrode location, electrocochleography

## Abstract

To preserve residual hearing during cochlear implant (CI) surgery it is desirable to use intraoperative monitoring of inner ear function (cochlear monitoring). A promising method is electrocochleography (ECochG). Within this project the relations between intracochlear ECochG recordings, position of the recording contact in the cochlea with respect to anatomy and frequency and preservation of residual hearing were investigated. The aim was to better understand the changes in ECochG signals and whether these are due to the electrode position in the cochlea or to trauma generated during insertion. During and after insertion of hearing preservation electrodes, intraoperative ECochG recordings were performed using the CI electrode (MED-EL). During insertion, the recordings were performed at discrete insertion steps on electrode contact 1. After insertion as well as postoperatively the recordings were performed at different electrode contacts. The electrode location in the cochlea during insertion was estimated by mathematical models using preoperative clinical imaging, the postoperative location was measured using postoperative clinical imaging. The recordings were analyzed from six adult CI recipients. In the four patients with good residual hearing in the low frequencies the signal amplitude rose with largest amplitudes being recorded closest to the generators of the stimulation frequency, while in both cases with severe pantonal hearing losses the amplitude initially rose and then dropped. This might be due to various reasons as discussed in the following. Our results indicate that this approach can provide valuable information for the interpretation of intracochlearly recorded ECochG signals.

## Introduction

For several years only patients without any residual hearing could receive a cochlear implant (CI). With ongoing clinical experiences and technical improvements, indication criteria were extended toward patients with residual hearing, typically in the low frequency range. Studies showed large benefits for the hearing ability of the patient when combining acoustic and electric stimulation (Baumgartner et al., 2007; Büchner et al., 2017; Gantz et al., 2005, 2018; Helbig et al., 2011; Incerti et al., 2013; James et al., 2005; Lenarz et al., 2009; Roland Jr et al., 2018; Von Ilberg et al., 2011). Special electrodes for demanding cochlear conditions were developed early on (Dhanasingh & Hochmair, 2021), but since the early 2000s electrodes were designed that aimed for atraumatic insertions and therewith preservation of residual hearing (Adunka et al., 2004; Lenarz et al., 2020; Risi, 2018). Also the surgical techniques were improved (Giordano et al., 2014; Khater & El-Anwar, 2017; Lehnhardt, 1993; Sikka et al., 2017). Over time different electrode lengths were designed with the aim to best match the breakpoint in the audiogram where the natural hearing of the patient drops down (Büchner et al., 2017; Dhanasingh & Hochmair, 2021; Lenarz et al., 2019). Later, it was detected that the cochleae of each individual patients have different geometries and that the differences especially in the length of the cochlea have to be regarded together with the audiogram when choosing the optimal electrode length and insertion depth for the individual patient (Erixon et al., 2009; Timm et al., 2018; Würfel et al., 2014). Over the years, a need for individualized cochlear implantation has been increasingly recognized (Dhanasingh & Hochmair, 2021; Lenarz et al., 2019).

This goes along with monitoring the state of the cochlea during insertion. The main goals are the preservation of hearing and cochlea structure as well as supporting the individual electrode position. In case of a deterioration of the recorded signal the surgeon could adapt his insertion technique, for example, by pausing the insertion or pull the electrode back in order to achieve recovery of the amplitude (Buechner et al., 2022). An electrophysiologic method that has been increasingly used for monitoring the state of the cochlea during electrode insertion is the recording of electrocochleography (ECochG; for reviews see, e.g., Barnes et al., 2021; Kim, 2020). Therewith the electric activity in the inner ear can be recorded intraoperatively during the insertion of the electrode. The recorded ECochG signal comprises of several components (Buechner et al., 2022; Eggermont, 1974; Forgues et al., 2014; Snyder & Schreiner, 1984). Cochlear microphonics (CMs) take place synchronous to the stimulus, therewith reflecting movements of the basilar membrane. Other components of the ECochG signal are summating potentials (SPs). SPs are a complex response similar to the stimulus envelope where the origin is not fully clear with contributions from inner and outer hair cells as well as the auditory nerve. Further components of the ECochG signal are action potentials (APs) and compound action potentials (CAPs), regarded to represent the response to onset and offset of the auditory nerve to acoustic stimuli. APs are produced by single fibers of the auditory nerve, whereas CAPs are the summated APs of all fibers. Another important component of ECochG are auditory nerve neurophonics (ANNs) which are a phase locked response to auditory stimuli and therewith regarded to reflect the status of the auditory nerve fibers occurring especially in the low frequency range. Unfortunately, especially in the low frequency range CMs and ANNs are difficult to separate from each other and are often referred to as ongoing responses (ORs).

Especially CAPs were investigated and were found to possibly be a more sensitive indicator for hearing loss than CMs (Adunka et al., 2010; Helmstaedter et al., 2018). Unfortunately patients with typical hearing losses usually suffer more from a hearing loss in the high frequency range, whereas CAPs can be recorded more reliable in the high frequency range than in the low frequency range. A limitation of all intraoperative recordings is the fact that the CI insertion can induce postoperative inflammatory processes (Seyyedi & Nadol Jr, 2014). Thus, it is still possible that in the case of a stable ECochG signal the hearing is preserved at the end of the surgery, but deteriorates afterward. Also, there are more physiological structures necessary for hearing than the cochlea itself (Haumann et al., 2019).

The stimulation for the ECochG measurement is usually done acoustically via an insert earphone placed in the outer ear canal using tone bursts of different frequencies and clicks. There are mainly two approaches for recording. In one approach the recording takes place extracochlearly which means that there is a recording electrode placed at or close to the promontory wall and usually connected to a clinical ABR device for stimulating and recording (Adunka et al., 2006; Choudhury et al., 2012; Dalbert et al., 2018; Fitzpatrick et al., 2014; Harris et al., 2011; Haumann et al., 2019, 2024; Mandalà et al., 2012; Suntinger et al., 2022). This electrode locally stays in the same spot all time during the complete CI electrode insertion. This is also the main advantage of the approach. If the recorded signal changes, it can be assumed those changes occur due to an intracochlear (IC) event. This could be for example a temporary blockage of the basilar membrane, or it could be indeed a trauma to the cochlea. Several research groups investigated the method, but most of them stated that a large trauma is likely to be detected, but a preserved ECochG signal not necessarily mean that also the residual hearing of the patient was preserved (Adunka et al., 2016; Dalbert et al., 2015, 2016; Haumann et al., 2024; Radeloff et al., 2012). On the other hand, few other studies detected significant correlations to residual hearing (Dalbert et al., 2018; Fitzpatrick et al., 2014; Suntinger et al., 2022) or speech perception (Abbas et al., 2017). Lately, there was an increasing trend toward IC recordings (Buechner et al., 2022; Calloway et al., 2014; Campbell et al., 2015; Dalbert et al., 2018; Haumann et al., 2019; Koka & Litvak, 2017). The recordings are performed via the electrode contacts of the electrode array, thus much closer to the relevant structures in the cochlea, yielding by far larger amplitudes. At first there were approaches where the IC signal was recorded using the CI electrode together with additional invasive recording equipment (Giardina et al., 2019; O'Connell et al., 2017), or there was a separate recording electrode temporarily inserted into the cochlea (Calloway et al., 2014; Dalbert et al., 2021). Later, integrated solutions were developed by the CI manufacturers where the IC recordings can be conducted using the clinical CI electrode steered telemetrically using the clinical stimulation coil and comfortable hard- and software solutions. In most cases the most apical stimulation contact is used for recording. The main disadvantage of this approach is that the recording site moves during CI electrode insertion. Thus, there is no comparison between the recorded amplitude before and after CI electrode insertion possible. Also, the ideal behavior of the signal amplitude is not yet clear. Several studies investigate the course of the recorded amplitude during CI electrode insertion and their relationship to the later hearing preservation (Buechner et al., 2022; Harris et al., 2017; Lenarz et al., 2022; Ramos-Macias et al., 2019). Some studies (Dalbert et al., 2018; Lenarz et al., 2022; O’Leary et al., 2020) detected a significant correlation between a drop in the IC ECochG signal amplitude and the long-term course of the hearing loss, whereas other studies (Giardina et al., 2019; Sijgers et al., 2021) postulated to include phase, latency, and neuronal components into the prediction of the postoperative hearing loss. Also, there are differences detected whether a drop in the ECochG signal amplitude occurs in the first or the second half of the insertion. Drops occurring during the first half could correspond to transient disruptions, whereas drops occurring in the second half of the insertion could correspond to hearing loss (Koka et al., 2018; Sijgers et al., 2021).

A late drop of the ECochG signal amplitude during electrode insertion could indicate trauma to the cochlear structures. But especially with long electrode arrays or stimulation with high frequencies, it would also be possible for the CI electrode contact used for recording to cross the generator of the response to the stimulation frequency, which would further increase the distance to the generator again. Saoji et al. (2019) introduced a new stimulation scheme and stimulated simultaneously with four different frequencies (500, 1000, 2000, and 4000 Hz) and recorded intracochlearly with the most apical CI electrode contact with one patient in order to operate as close as possible to at least one generator all the time during insertion. This scheme was applied on a larger group of 10 patients (Saoji et al., 2023) where the recordings were done during and after CI electrode insertion. Based on the electrophysiological data, it was concluded that in some cases the frequency generator was presumably crossed and that the results from other measurement contacts should also be used to distinguish whether an amplitude drop is caused by this or by a trauma. Dalbert et al. (2021) and Sijgers et al. (2021) recorded simultaneously extra and intracochlearly, Dalbert et al. (2021) with a special IC recording electrode and Sijgers et al. (2021) using the CI electrode itself which was connected to a clinical ABR device. They compared the course of the extra- and intracochlearly recorded ECochG signals in order to combine the advantages of both methods.

Still, the cochlea is a kind of black box. With X-ray fluoroscopy, for example, the insertion process can be observed (Fishman et al., 2003), but not the function of the cochlea at the corresponding frequency. Also, they use radiation which is a clear disadvantage. Several research groups also investigated together with clinical imaging whether a deviation of the electrode array into the scala vestibuli could be detected by recording ECochG. Koka et al. (2018) investigated this with 32 patients where seven deviations took place. All seven were detected, but six out of 25 correctly inserted electrodes were wrongly identified as deviation. O'Connell et al. (2017) did not detect a clear relation with 16 patients, but of course, the later speech perception was worse than in cases without electrode deviations. Baumhoff et al. (2022) investigated whether intracochlearly recorded SP could be used for estimating the position of the electrode in the cochlea in guinea pigs. The evaluation was done using a µCT. Together with a frequency estimation using a modified Greenwood function they postulate a reliable estimation of the electrode position.

To summarize, there has been a lot of research regarding intracochlearly recorded responses and how their behavior reflects the progress of the CI electrode insertion and the status of the cochlea in recent years. To better understand the changes in ECochG signals and whether these are due to the position of the electrode in the cochlea or to the trauma generated during insertion, the behavior of intracochlearly recorded ECochG signals intra- and postoperatively was analyzed retrospectively. The main focus of the current study was to relate the behavior to the presumed location of the frequency generators of the response to the stimulation frequency in six patients who received an MED-EL implant designed for hearing preservation.

## Methods and Materials

For CI users with residual hearing, intraoperative ECochG recordings are performed routinely in our clinic. In the present study data of six patients where intracochlear (IC) potentials were recorded using the CI electrode and the clinical software provided by the implant manufacturer was analyzed retrospectively. The recordings were performed intraoperatively during electrode insertion at different discrete insertion steps using the most apical CI electrode contact for recording. After insertion intraoperatively, as well as postoperatively after six months of CI use, the recordings were done using different CI electrode contacts. The recordings were correlated with the electrode location in the cochlea as well as pre- and postoperatively obtained PTA thresholds. The study was approved by the local ethics committee (approval number: 1897–2013) and is in accordance with the ethical standards of the Declaration of Helsinki.

### Preoperative Evaluation

The preoperative CI candidate evaluation is standardized at our clinic (Haumann et al., 2019) and includes subjective and objective audiometric evaluation as well as clinical imaging (CT and fMRI scan) and other examinations. The individual patient chooses an implant system according to his or her personal preferences, and the length of the electrode is determined together with the surgeon depending on residual hearing and length of the cochlea (Timm et al., 2018; Würfel et al., 2014).

The pure tone audiometry (PTA) was measured using an audiometer type AD2117 (earlier AD17 and AD2017) by Audio-DATA GmbH, Duvensee, Germany. The stimuli were calibrated to hearing level (HL). Air conduction stimuli were presented using headphones (HDA300, earlier HDA200, Sennheiser electronic GmbH, Wedemark, Germany) and bone conduction stimuli were presented using a bone transducer (KLH96, Westra Elektroakustik GmbH, Meitingen, Germany).

### IC ECochG Recordings Using the CI Electrode

#### Measurement Setup

For IC recordings different MED-EL (Innsbruck, Austria) FLEX electrode arrays were used with the clinical CI measurement setup for MED-EL implants (MAX-Box, coil, and laptop) and the clinical software (MAESTRO 7.0, MED-EL). The ECochG recordings were conducted via the ART task in the software and parameters were chosen according to our previous protocol (Haumann et al., 2019). The acoustic stimulation was delivered by a Nicolet Viking EDX system (Natus Medical Incorporated, Pleasanton, CA, USA) triggered by the MAESTRO Software. The stimulation was delivered using insert earphones (Nicolet TIP300, Natus Medical Incorporated, Pleasanton, CA, USA) and a sterile foam plug placed in the outer ear canal.

#### Intraoperative Procedures

During the CI electrode insertion IC potentials were recorded at different discrete insertion steps (after 3, 6, 9 and the final number of electrode contacts inserted as determined visually by the surgeon) using the most apical electrode contact (C1). In some of the cases, the electrode was intentionally not fully inserted, so the final number of electrodes inserted was not always 12. This concept of partial insertion is described in Lenarz et al. (2019). Especially with good residual hearing in the low frequency range the patient could receive a short CI electrode in order to maximally preserve residual hearing for best hearing outcome in electric-acoustic stimulation (EAS) (Büchner et al., 2017). However, if the residual hearing gets lost in the long term, the patient can only use the short electrode for electric stimulation with a potential reduction in performance (Büchner et al., 2017). To improve, the patient would have to undergo a full surgery for reimplantation. In case of partial insertion a long electrode is inserted only partly to a certain inserted electrode depth (IED) and such mimics a short electrode. In case of a deteriorating residual hearing the CI electrode can be inserted deeper in just a small revision surgery without using a new implant.

For the recordings, the following parameters were chosen (see also Haumann et al., 2019): A 19.4 ms recording window was used to get a clear view of the complete wave for better signal processing analysis. The repetition rate was 19.1 Hz, the polarity was set to rarefaction. The number of averaged trials per trace was set to 50, yielding a resulting recording time per trace of about 30 s. For stimulation a 500 Hz tone burst was applied (2 ms rise time, 4 ms plateau time, 2 ms fall time). The stimulus level was calibrated to normal hearing level (nHL). The stimulation level was set intraoperatively to at least to 20 dB above the air conduction threshold, in case of a more severe hearing loss to the maximum stimulation level of 99 dB nHL.

The CI electrode was inserted very slowly. The surgeon visually identified each defined discrete recording step, paused the insertion and one trace was recorded at C1. This was repeated until the electrode was inserted as deep as intentioned. After reaching the desired IED the IC potentials were recorded at different electrode contacts (C1, C3, C6, C9 [, C12]). The position of the electrode was routinely evaluated via cone beam CT scan, either intraoperatively or one day postoperatively.

### Postoperative Recordings

The so-called test switch-on (TSO) phase took place 1–3 days after surgery. Here the PTA was measured in air and bone conduction, a cone beam CT scan was performed (if not already done intraoperatively) and the implant was stimulated as a test. For PTA this work focuses on the 6-month (6M) appointment after 6 months of CI use. Here the PTA was measured via air conduction, but a bone conduction measurement is usually only done if the patient had an air bone gap before surgery which was not the case in any of the patients examined here.

The IC ECochG recording using different electrode contacts (C1, C3, C6, C9 [, C12]) was repeated at the 6M appointment. The parameters were set identically to the intraoperative measurements, however, the number of averaged trials per trace was set to 100, as measurement time was not as critical as during surgery. The stimulation was done with the Nicolet Synergy EDX system (Natus Medical Incorporated, Pleasanton, CA, USA). Postoperatively the stimulation level was set to the intraoperatively used level as a starting point, but the patient was asked to indicate on a visual analog scale whether the level was comfortable. If necessary, the level was adapted accordingly.

### Data Analysis

#### PTA Thresholds

The differences in the air conduction thresholds before surgery and after six months of CI use were tested for significance on a 5% level with a paired-sample t-test, and the correlation coefficients were calculated using the according MATLAB functions. Hearing preservation for the different IEDs was classified similarly to Suhling et al. (2016). The audiometric pure tone low frequency hearing threshold (PTA_low_) was calculated as mean of the air conduction thresholds at 250 Hz, 500 Hz, and 1000 Hz. In case of no response at the highest level, the response was set to 10 dB above the audiometer limit which is 105 dB HL at 250 Hz and 110 dB HL for 500 Hz as well as 1000 Hz. The postoperative hearing preservation was classified to yield a PTA _low_ shift between pre- and postoperative data up to 15 dB, a shift between 15 dB and 30 dB and a shift of more than 30 dB.

#### ECochG Recordings

The ECochG data was analyzed by using Fast Fourier Transform (FFT) using MATLAB R2017a (The Mathworks, Inc., Natick, Massachusetts, USA). The preprocessing of the recorded traces was performed in the same way than already described in Haumann et al. (2019). In short, the raw data was exported from the recording device and imported into MATLAB. There the signal was resampled to 119 kHz in order to meet the exact frequency bin in the FFT. The 500 Hz stimulus was comprised of 2 ms ramp up and down each and 4 ms plateau, resulting in a stimulus length of 8 ms which corresponds to four cycles. The signal part comprising the response which was also set to 8 ms (961 samples) was extracted and detrended in order to set the mean to zero. After that, the FFT was calculated on the processed data. For the FFT a hamming window of the same length (961 samples) was used, resulting to 481 frequency bins (including the zero bin) and a bin width of 125 Hz. The amplitude at the 500 Hz bin was used for further analysis.

The noise floor was estimated from earlier data (Haumann et al., 2019). The calculation is given in detail in the supplemental material of the current work. In short, recordings with 0 dB nHL stimulation level were done for each stimulation frequency and processed in the same way as the data obtained with higher stimulation. There were 10 patients included in the earlier study, yielding 10 data sets per appointment. The amplitudes of all bins in the FFT were separately averaged across the 10 data sets. This procedure was done for the intraoperative appointment and for a follow up appointment. With these means and standard deviations a 99% confidence interval was calculated as µ ± 2.576 × σ, separated for intraoperative and postoperative settings.

#### Determination of Electrode Location and Place Frequencies Inside the Cochlea

For each patient, OsiriX MD (Pixmeo SARL, Switzerland) was used to trace the cochlear lateral wall (LW) within the preoperative and the electrode array within the postoperative CT images. A detailed description of this tracing procedure is given in Timm et al. (2018). In order to determine the location of the electrode during insertion, the algorithm described in Schurzig et al. (2023) was used and is hence only described briefly in the following: based on the LW tracing, the 3D shape of the LW was reconstructed within the consensus coordinate system proposed in Verbist et al. (2010) and enhanced to a full reconstruction of the cochlear lumina using the IC cross-section derived in Schurzig et al. (2021). The electrode array and insertion angle specific average distance between LW and array (see also Salcher et al., 2021) were then used to estimate the path of the array inside the scala tympani of the patient specific 3D cochlear reconstruction. Finally, the array was virtually inserted into cochlear reconstruction along the derived insertion path such that 3, 6, or 9 contacts were located inside the cochlea, respectively. For determining the final location of the electrode array after insertion, the postoperative tracing of the electrode array was registered to the LW using the HelReg method described in Schurzig et al. (2018). The tonotopic place frequencies at the locations of the individual contacts were determined based on the Organ of Corti frequency mapping approach proposed by Helpard et al. (2021).

### Subject Demographics

The data of six patients included was analyzed (43–72 yrs, Ø 60 yrs, one male, five female). All received a MED-El synchrony implant with different electrode lengths and different IEDs. Their details are given in Table 1.

**Table 1.** Details of the Subjects and the Implanted Electrodes.

ID	Electrode	Inserted electrode depth (mm)	Age at surgery (yrs)	Sex	Side	Duration of hearing impairment (yrs)	Etiology
**S01**	Flex28	26.6	71.9	f	r	10	Unknown/progressive
**S02**	Flex20	17.3	42.7	f	r	15	Sudden hearing losses
**S03**	Flex28	22.1	43.3	f	r	10	Unknown/progressive
**S04**	Flex28	19.6	75.3	f	l	14	Unknown/progressive
**S05**	Flex28	21.4	56.7	f	l	20	Sudden hearing losses/progressive
**S06**	Flex28	26.1	71.9	m	r	3	Unknown/progressive

## Results

### Hearing Preservation as Measured by PTA

**Figure 1.**
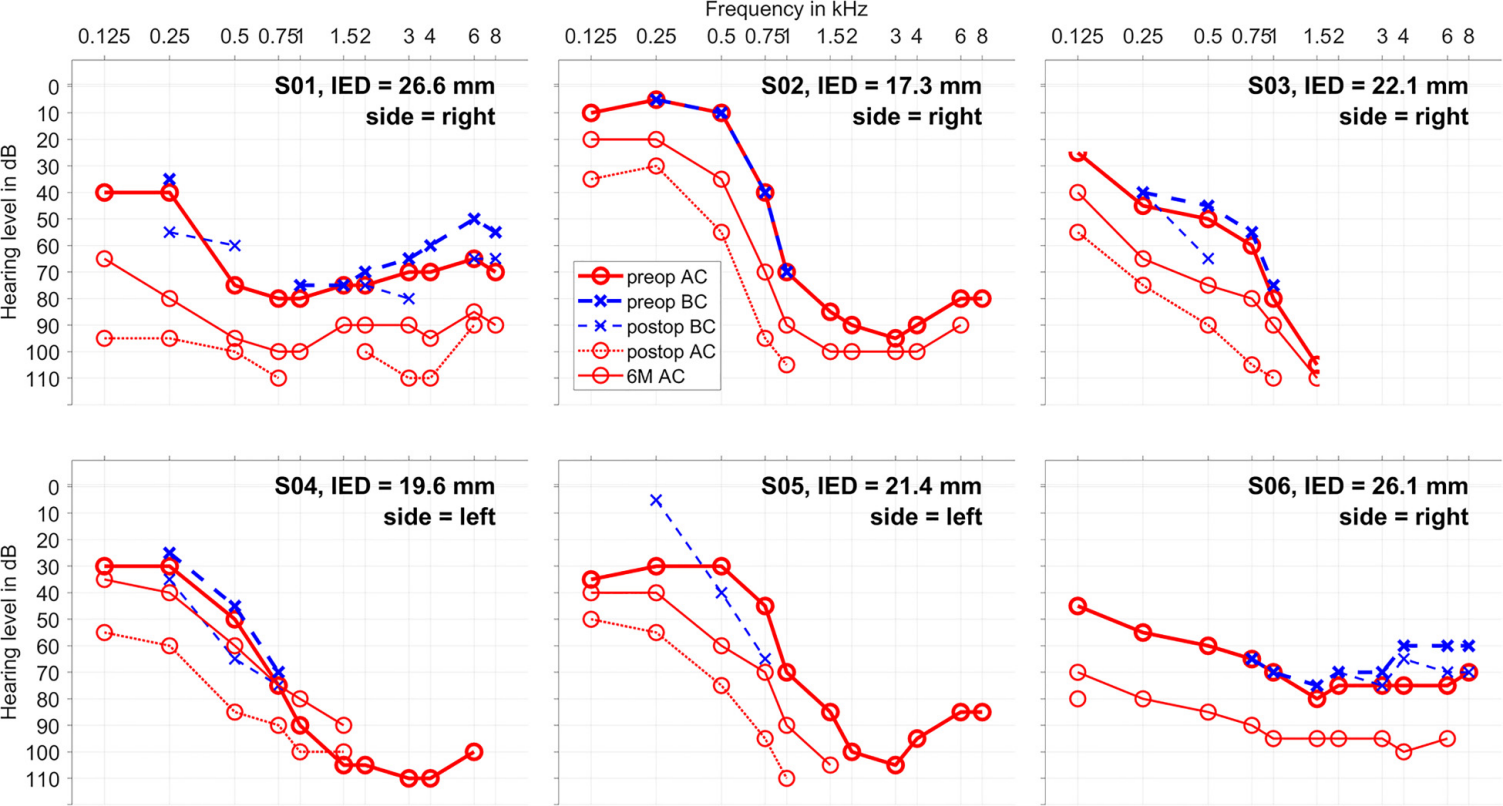
Audiograms: air conduction (AC, in red) and bone conduction (BC, in blue) for each patient together with the inserted electrode depth and the implanted side. The measurements before surgery are shown in thicker lines, the measurements 1–3 days after surgery in dashed lines and the measurements after six months in thinner lines (only AC).In S05 no BC was measured on the explicit preoperative appointment, but earlier audiograms show no air-bone-gap. In S02 no postop BC could be measured due to pain.

The audiograms of all *n* = 6 individuals are given in Figure 1. The hearing preservation as measured by PTA is classified in Table 2. The tympanogram was performed only preoperatively, and in all six cases it was unremarkable, not indicating liquid or negative pressure in the middle ear.

**Table 2.** Hearing Preservation Classified by Low Tone Air Conduction Threshold Shifts Before Surgery and at 6M Appointment, Similar to Suhling et al. (2016). The Low Frequency PTA Was Calculated as Mean of the Threshold at 250, 500, and 1000 Hz.

	PTA _low, 6 month_—PTA _low, pre_		
	Δ PTA _low_ ≤ 15 dB	15 dB ˂ Δ PTA _low_ ≤ 30 dB	Δ PTA _low_ ˃ 30 dB
**IED ≤ 20** ***n*** = 2	1 50%	1 50%	0 0%
**20 < IED ≤ 24** ***n* = 2**	0 0%	2 100%	0 0%
**24 < IED ≤ 28** ***n* = 2**	0 0%	2 100%	0 0%

The individual audiometric threshold shifts pre–post surgery are given in Table 3. The mean (*n* = 6) audiometric threshold shift in the low frequency range (PTA_low_) between preoperative and 6M appointment was significant with a correlation coefficient of *r* = .87*.

**Table 3.** Inserted Electrode Depth (IED), Audiometric Thresholds PTA _low_ Before Surgery and at 6M and Hearing Preservation Group (HP Group) Classified by Audiometric Threshold Shifts According to Suhling et al. (2016).

ID	IED (mm)	PTA _low_ (dB HL) before surgery	Preop transtymp ECochG threshold (dB nHL)		6M	
			CM 2 kHz	CAP click	PTA _low_ (dB HL) 0.25–1 kHz	HP group
**S01**	26.6	65.0	90	nr	91.7	15–30 dB
**S02**	17.3	28.3	declined by patient		48.3	15–30 dB
**S03**	22.1	58.3	80	nr	76.7	15–30 dB
**S04**	19.6	56.7	80	nr	60.0	0–15 dB
**S05**	21.4	43.3	90	nr	63.3	15–30 dB
**S06**	26.1	61.7	80	80	86.7	15–30 dB

nr = no responses.

### Intracochlearly Recorded ORs—Example Case

**Figure 2.**
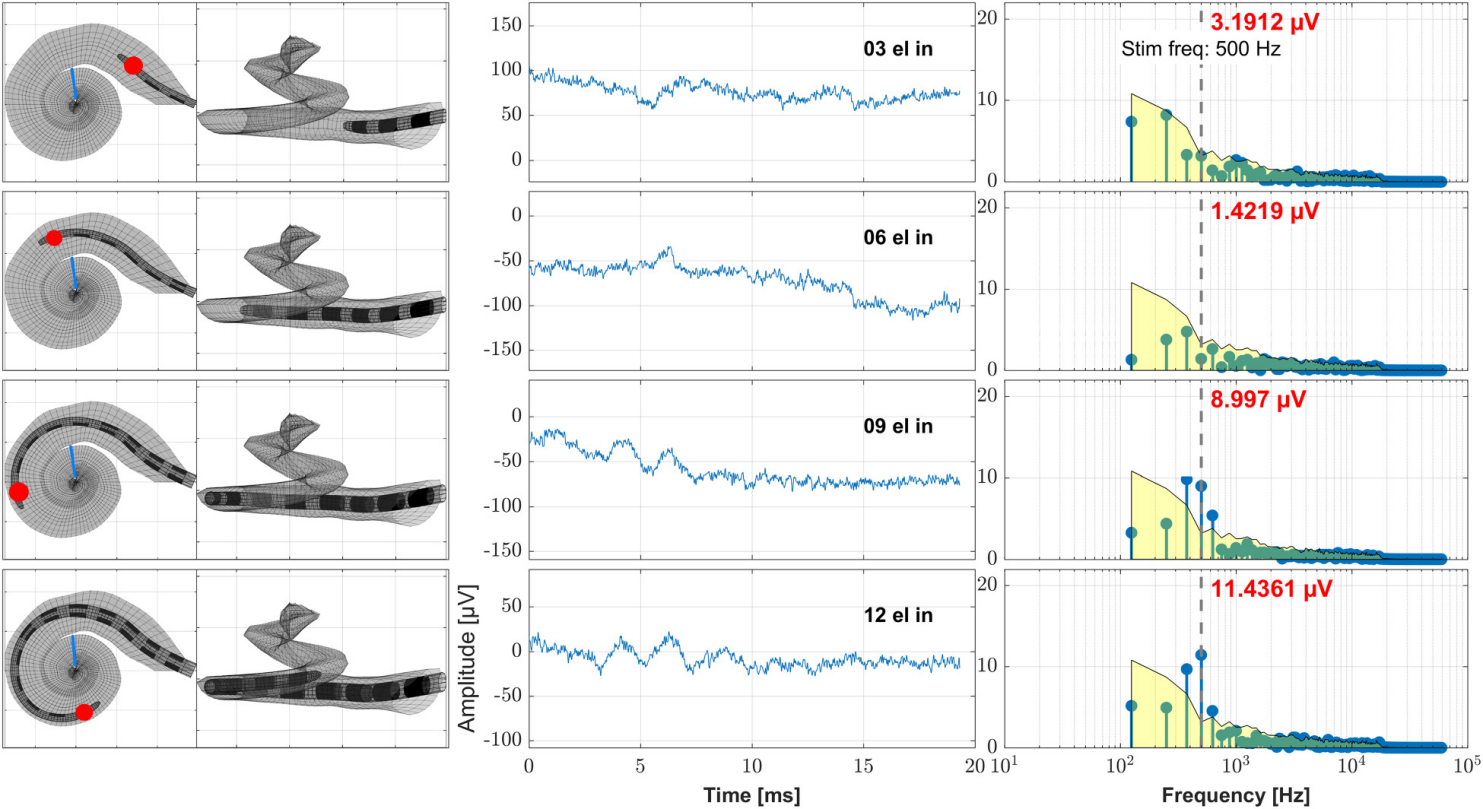
Example case (subject S02, female, age 42.7 years, Flex20, 17.3 mm inserted electrode depth, right side). Here the data obtained during the cochlear implant (CI) electrode insertion is shown. The recordings took place at discrete electrode insertion steps using the most apical contact C1. On the left panels these steps are visualized by means of the assumed electrode insertion depth as calculated from the preoperative clinical imaging data with the recording contact being highlighted (Schurzig et al., 2023). The blue line represents the location in the cochlea where the 500 Hz characteristic frequency region is located. On the middle panels the time signal of the ongoing response is shown and on the right panels the frequency analysis by Fast Fourier Transform (FFT). The red number at each FFT plot states the amplitude at the frequency bin of the stimulus frequency (500 Hz), the gray vertical dashed line represents the stimulus frequency (500 Hz) and the yellow patch visualizes the noise floor. From top to down the ongoing CI electrode insertion is shown (recording after 3, 6, 9, and 12 electrodes inserted).

**Figure 3.**
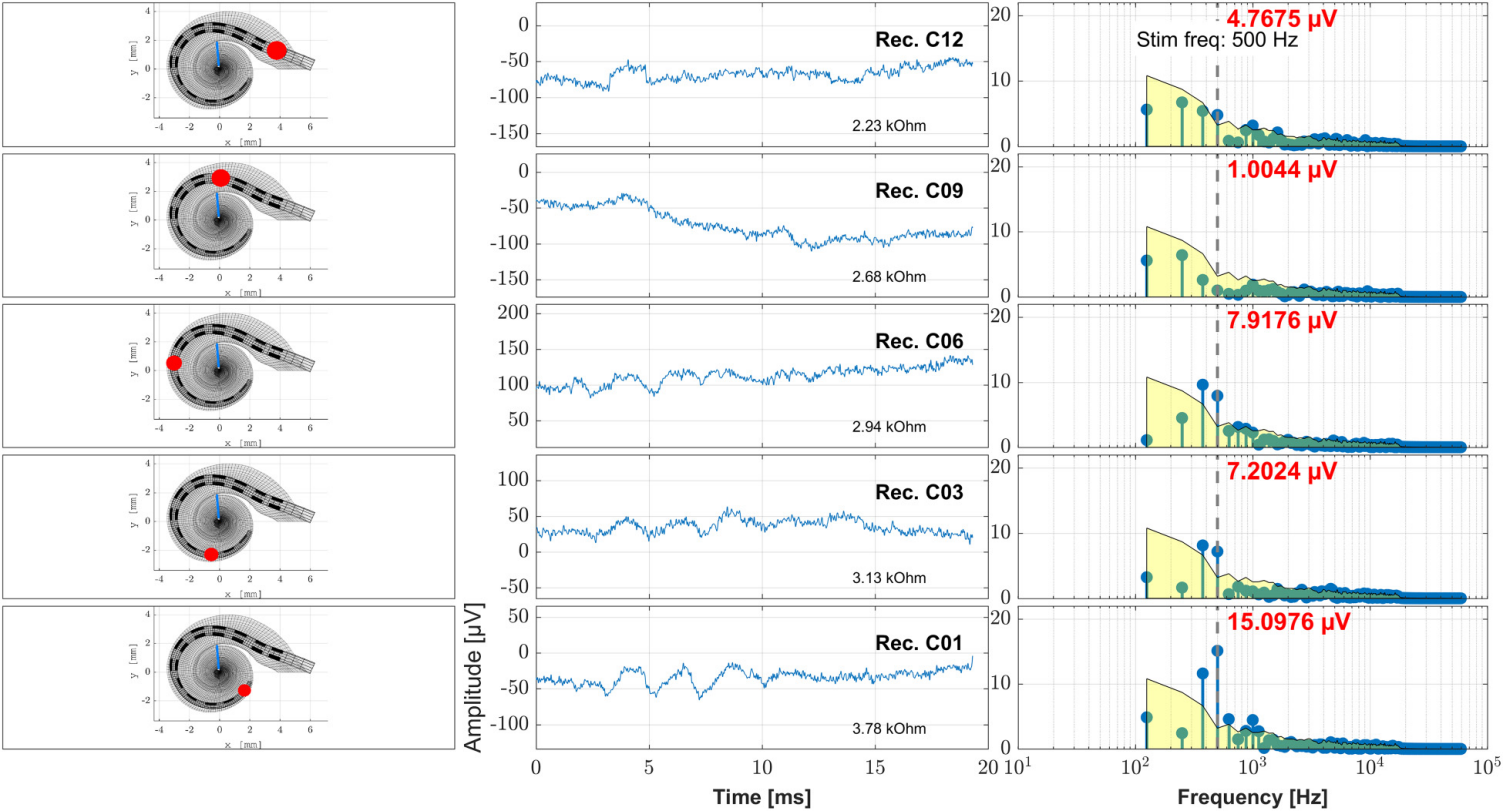
Example case (subject S02, female, age 42.7 years, Flex20, 17.3 mm inserted electrode depth, right side). Here the intraoperative data obtained directly after electrode insertion is shown where the recording was performed at different recording contacts. On the left panels the position of the electrode in the cochlea as measured from the postoperative clinical imaging data is shown with the current recording contact being highlighted. The blue line represents the location in the cochlea where the 500 Hz characteristic frequency region is located. On the middle panels the time signal of the ongoing response is shown and on the right panels the frequency analysis by Fast Fourier Transform (FFT). The red number at each FFT plot states the amplitude at the frequency bin of the stimulus frequency (500 Hz), the gray vertical dashed line represents the stimulus frequency (500 Hz), and the yellow patch visualizes the noise floor. From top to down the data obtained at different recording contacts is shown (12, 9, 6, 3, 1). Also the impedance of each recording contact is given.

**Figure 4.**
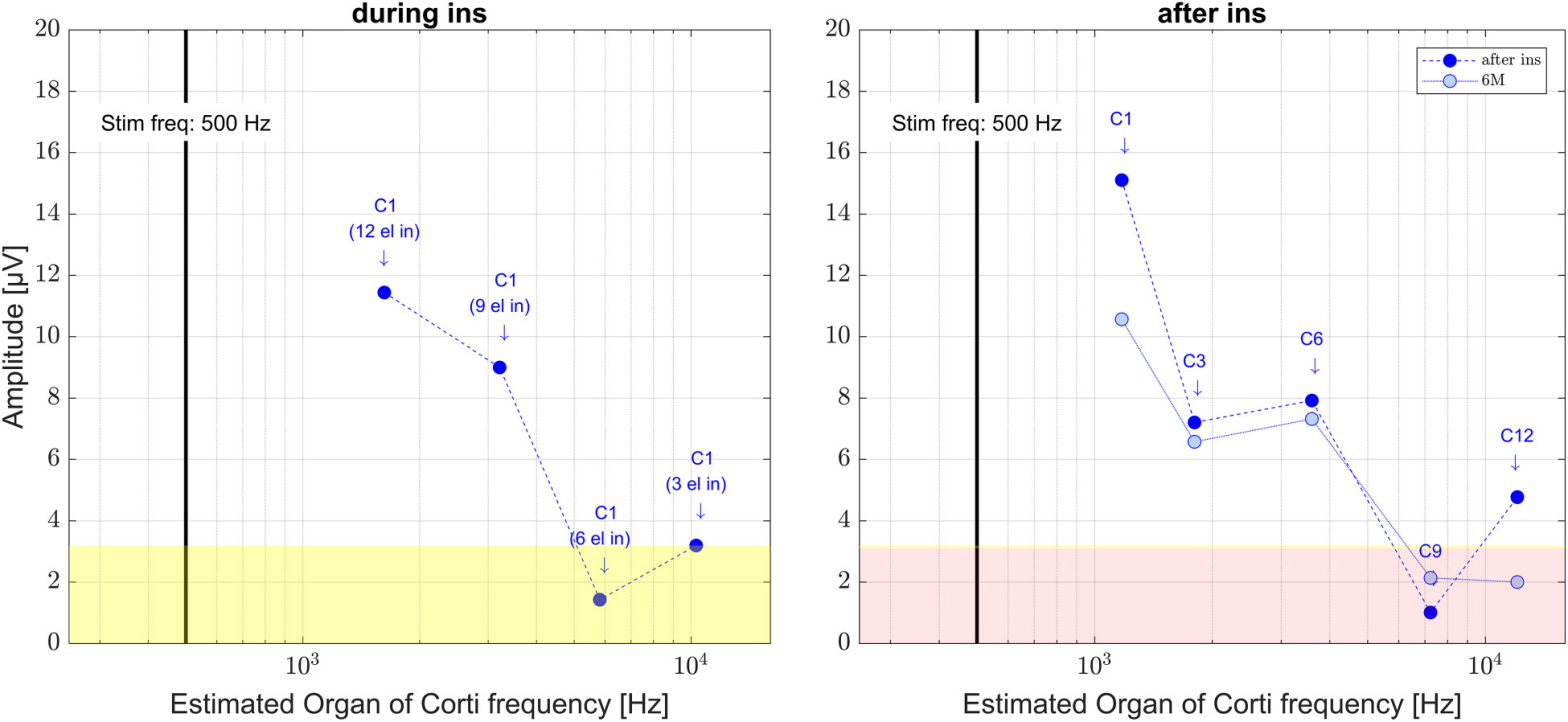
Recorded OR amplitudes of an example case (subject S02, female, age 42.7 years, Flex20, 17.3 mm inserted electrode depth, right side). On the *x*-axis the estimated Organ of Corti (OC) frequency is given, on the *y*-axis the amplitude of the 500 Hz bin in the Fast Fourier Transform. The vertical bar represents the stimulation frequency of 500 Hz. The stimulation level was 70 dB normal hearing level (nHL) for all measurements. The left panel shows the data during insertion. The ongoing responses (ORs) were recorded at C1 at different insertion steps and the OC frequency was estimated model-based using preoperative imaging data (Schurzig et al., 2023) and a frequency mapping (Helpard et al., 2021). The right panel shows the data recorded intraoperatively after insertion as well as at the 6M appointment. Here the ORs were recorded at different electrode contacts. The position of the electrode in the cochlea was measured using registered preoperative and postoperative imaging data (Schurzig et al., 2018). The yellow patches visualize the intraoperative noise floor and the light red patch the postoperative noise floor for the frequency bin at 500 Hz in the Fast Fourier Transform of the 0 dB recordings in earlier data.

At first, the individual data of subject S02 is shown in detail as an example case (Figures 2–4). This patient received a Synchrony implant with fully inserted Flex20 electrode at her right side. In Figure 1, the course of her audiogram is shown before surgery, one day postop at the TSO and at the 6M appointment, showing a normal hearing ability in the low frequencies up to 500 Hz and a steep drop down toward the high frequencies. The air conduction showed a drop at the TSO, but on the long term (6M) the hearing was mainly preserved.

In Figure 2, the intraoperative ECochG recordings during insertion are shown together with the model-based estimated position of the recording electrode in the cochlea and a frequency mapping. With ongoing insertion progress, the amplitude of the OR rose until the recorded maximum was reached at full electrode insertion.

In Figure 3, the intraoperative ECochG recordings directly after insertion are shown together with the measured position of the recording electrode in the cochlea and an estimated frequency mapping. With recording deeper in the cochlea, the amplitude of the OR rose until the recorded maximum was reached at the deepest electrode contact.

Figure 4 shows the recorded OR amplitudes allocated to the OC frequency at the position of the electrode in the cochlea. Here, it can be shown that the amplitude of the OR rose until the recording was performed deepest in the cochlea. This could be observed for all measurements in this case: during insertion, after insertion and for the 6M appointment respectively.

### Intracochlearly Recorded OR—Group Data

The six cases were grouped by the IED which was chosen according to the preoperative audiogram. There were two cases with short IED (IED ≤ 20 mm), two cases with medium IED (20 mm < IED ≤ 24 mm) and two cases with long IED (24 mm < IED ≤ 28 mm). In all cases, intraoperative IC OR responses could be detected in the cochlear spiral. In the four cases with short and medium IED the amplitudes rose monotonously with ongoing electrode insertion, whereas in the two cases with long IED the amplitude rose with ongoing electrode insertion but dropped within the second half of the insertion. At both postinsertion measurements, intraoperatively after insertion as well as during the follow up (6M), the recording was repeated on different electrode contacts throughout the cochlea. Here the results were similar to the intraoperative measurements. In the four cases with short and medium IED the recorded amplitude rose with going deeper into the cochlea, whereas in the two cases with long IED the amplitude firstly rose, but deeper in the cochlea it dropped.

**Figure 5.**
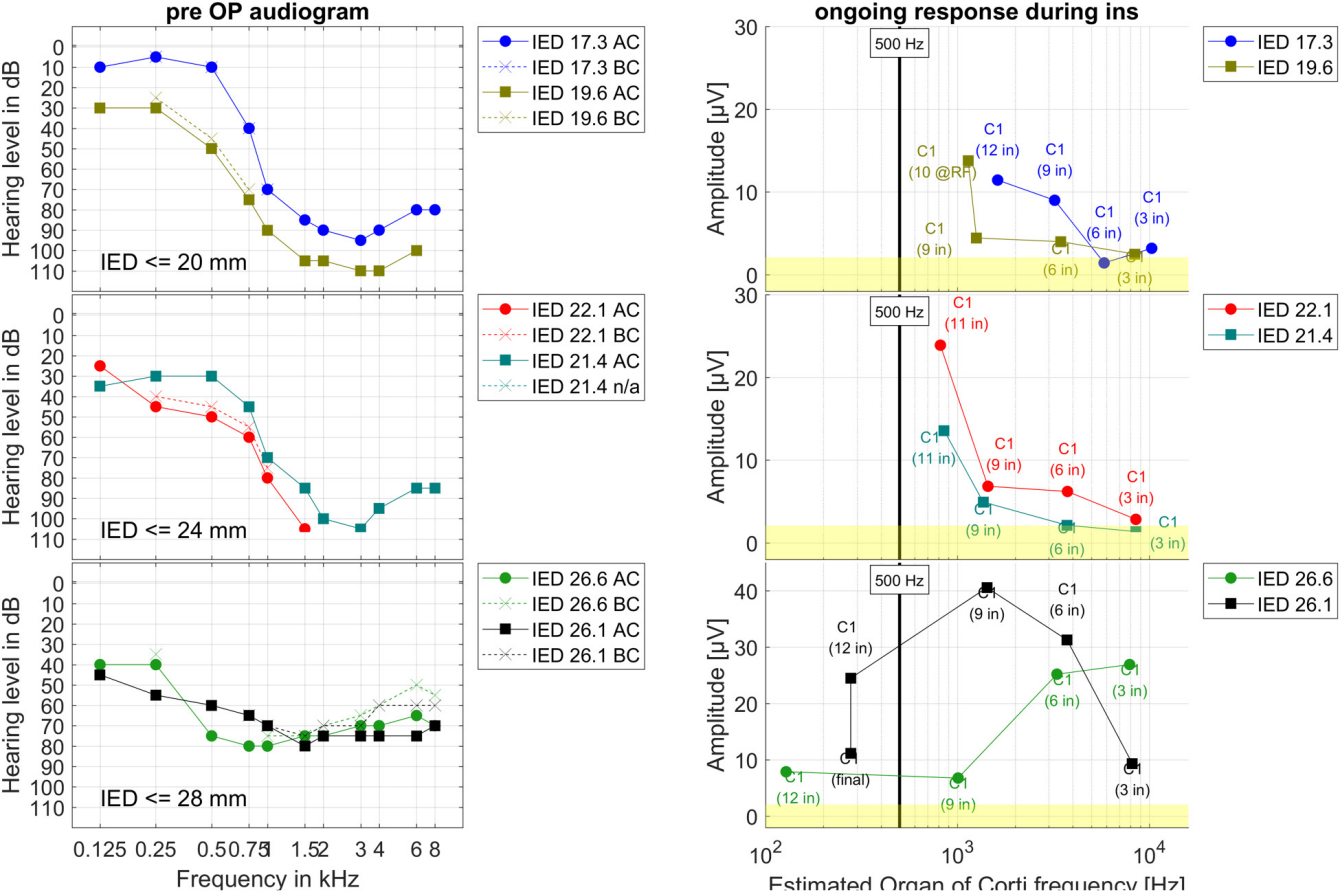
Group data, clustered by inserted electrode depth (IED), IED ≤ 20 mm, 20 mm < IED ≤ 24 mm, 24 mm < IED ≤ 28 mm, two patients each. On the left panels the audiograms before surgery are shown. On the right panels the amplitudes at 500 Hz of the Fast Fourier Transform (FFT) of the ongoing response (OR) recorded at C1 during the CI electrode insertion are drawn over the estimated frequency of the organ of Corti in the cochlea. The vertical bar represents the stimulation frequency of 500 Hz. The yellow patches visualize the intraoperative noise floor for the frequency bin at 500 Hz in the FFT of the 0 dB recordings in earlier data.

In Figure 5, the audiograms before surgery and the intraoperatively recorded OR amplitudes during electrode insertion are shown allocated to the OC frequency for all six patients. The allocations were estimated using the model based estimated position of the recording electrode in the cochlea and a frequency mapping.

**Figure 6.**
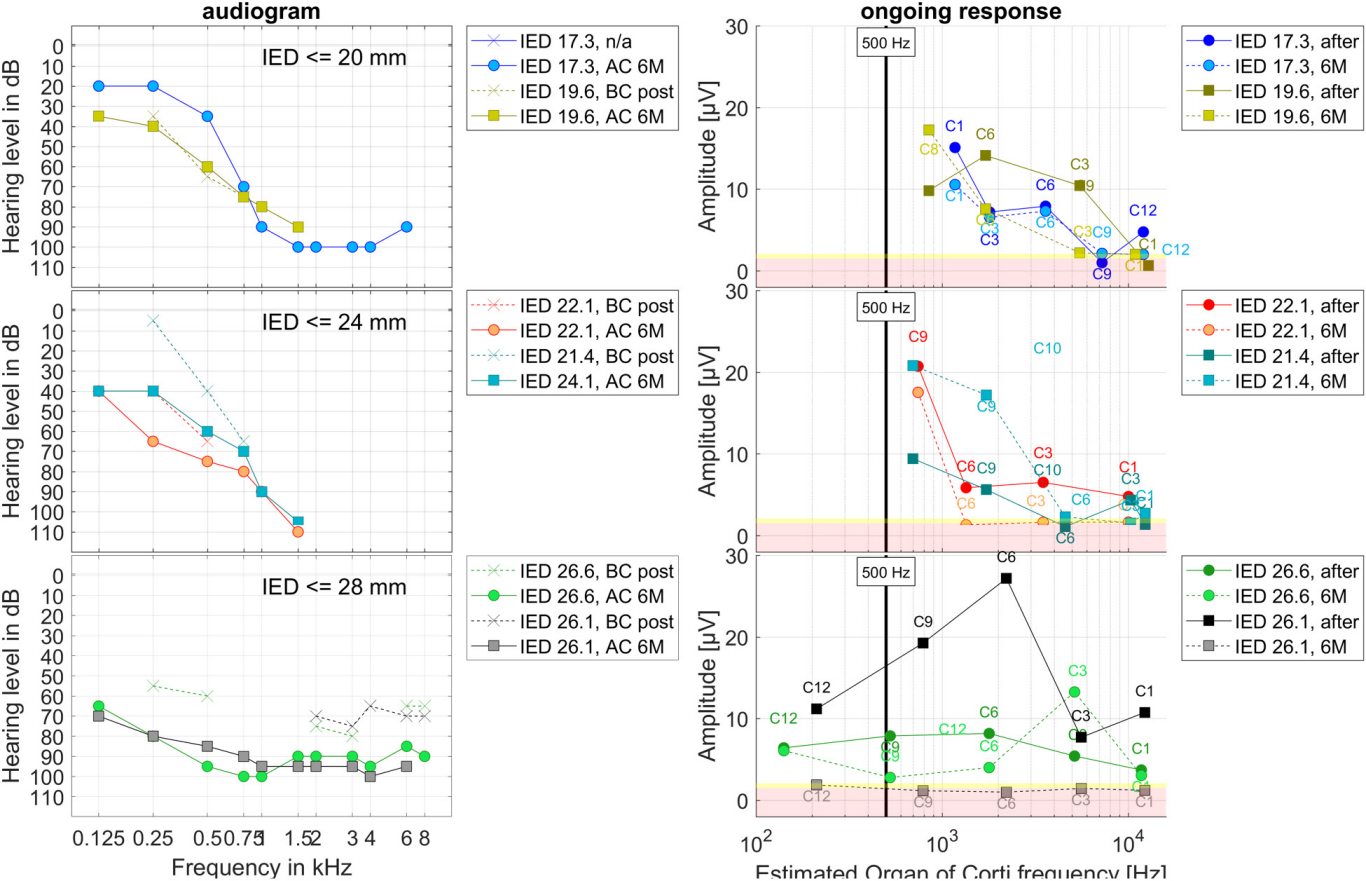
Group data, clustered by inserted electrode depth (IED), IED ≤ 20 mm, 20 mm < IED ≤ 24 mm, 24 mm < IED ≤ 28 mm, two patients each. On the left panels the postoperative BC audiograms taken 1–3 days after surgery and the AC audiograms at 6M are shown. On the right panels the intraoperative data directly after electrode insertion (full line) as well as at 6M (dashed line) where the recording was performed at different recording contacts is drawn about the estimated frequency of the Organ of Corti in the cochlea. The vertical bar represents the stimulation frequency of 500 Hz. The yellow patches visualize the intraoperative noise floor and the light red patches the postoperative noise floor for the frequency bin at 500 Hz in the Fast Fourier Transform of the 0 dB recordings in earlier data.

In Figure 6, the postoperative BC audiograms taken 1–3 days after surgery and the AC audiograms at 6M as well as the recorded OR amplitudes intraoperatively after electrode insertion and at 6M are shown allocated to the OC frequency for all six patients. The allocations were estimated using the calculated position of the recording electrode in the cochlea and a model based frequency mapping.

## Discussion

In this study six data sets of patients where IC ECochG was recorded intraoperatively during and after CI insertion as well as postoperatively after six months of CI use were analyzed retrospectively. These data were combined with a calculation of the estimated electrode position in the cochlea as well as the presumed according frequency generator. Furthermore, the data was related to the residual hearing preservation.

All subjects were provided with a CI electrode aiming for hearing preservation surgery according to our clinical standards. The IED was chosen according to the residual hearing of the subjects. It is known that a long electrode insertion has a high risk of damage to the cochlea (Suhling et al., 2016). On the other hand, a patient with a short electrode without useable acoustic hearing in the low frequency range has a low speech perception (Büchner et al., 2017), so the IED was carefully chosen for the individual patient.

At the TSO appointment 1–3 days after surgery, PTA measurements are not performed for precise residual hearing estimation. They are performed to determine whether any residual hearing could be preserved, and if necessary to counteract with medication. Quite often there are some acute effects in the middle and inner ear (e.g., liquid entrapment in the middle ear) which lead to a transient deterioration of the air conduction threshold. It can be concluded that no large trauma occurred during surgery, as in all cases there was still measurable hearing on the long term. Nevertheless, it is noticeable that BC thresholds postoperatively are near to preoperative thresholds, while air conduction thresholds are lower after six months. Ultimately, this could come from postoperative inflammatory processes or a progressive underlying disease responsible for the hearing loss leading to CI surgery.

The preservation of the residual hearing in the low-frequency range with air conduction was classified into three groups similar to Suhling et al. (2016). After 6 months of CI use S04 with an IED of ≤ 20 mm was classified to the good hearing preservation group (0–15 dB) and all other patients to the medium group (15–30 dB). These results are in line with the findings of Suhling et al. (2016). Here 50% of the patients receiving a 20 mm IED were in the good HP group and 50% in the medium or worst group which is the case in our data. For our two patients receiving a 20 mm < IED ≤ 24 mm compared to Suhling et al. (2016) one of the two patients should be in the good HP group and the other one in the medium HP group, but both were in the medium HP group. For our two patients receiving a > 24 mm IED compared to Suhling et al. (2016) one of the two patients should be in the medium HP group and the other one in the worst HP group, but in our data, both were in the medium HP group. Nevertheless, the significance of two patients in each group is very limited. Especially with limited residual hearing already preoperatively the validity of the worst hearing preservation group (≥ 30 dB) is limited, as in these cases the recorded pure tone threshold comes close to the audiometer limit. Thus, not in all cases a hearing loss of 30 dB or more can be detected with the audiometer.

One of the largest general drawbacks of IC recordings during CI electrode insertion via the electrode contacts is that the recording site moves which was for example reported by Calloway et al. (2014). Thus, it is currently not known how the recorded signal should behave for optimal hearing preservation. In theory the OR amplitude should rise with ongoing electrode insertion. But if there is a drop in the amplitude it could, for example, be caused by changes in location of the recordings site, a contact and mechanical damping of the basilar membrane, or by an acute cochlear trauma which would probably be accompanied by a loss of residual hearing. So, the behavior of the intracochlearly recorded OR amplitude is currently being investigated by several research groups.

One aspect that has a major influence on the results is the number of averaged trials per trace used for the measurements. For smooth data with small variability, a lot of trials have to be performed for each trace (e.g., Suntinger et al., 2022 recorded 400 responses per frequency). Our approach was different as we did not want to overly stress surgical time and were aiming to implement the measurements in everyday clinical practice. We therefore decided to carry out comparatively few averaged trials per trace (*n* = 50 intraop and *n* = 100 in the follow up) in order to remain within a clinically acceptable recording time. During the surgery, the potentials were visually evaluated by experienced audiologists in order to immediately provide the surgeon with quick qualitative feedback. Postoperative, quantitative analysis revealed a deviation in results compared to the acute interpretation. This emphasizes the inaccuracy of the intraoperative interpretation of raw data and speaks for the need of real time data evaluation.

One limitation in our noise calculation is that it was performed with 10 data sets from an earlier study. Consequently, the recorded sessions are not fully comparable. In the previous study (Haumann et al., 2019), we calculated the noise based on 0 dB stimulation traces. We did not repeat this evaluation here for the same reasons as mentioned above: to not stress surgical time with additional measurements. This resulted in a few outlying datasets where especially at low frequencies the energy in the frequency bin clearly exceeds the noise level in the low frequencies (e.g., subject S01 or S04, both intraop after insertion, see supplemental material). However, this seems to be a common issue as previous studies also reported those issues even when using a larger amount of averaged trials per trace (Suntinger et al., 2022; Walia et al., 2022). At the time of measurement, the recordings during insertion via the electrode contacts could only be performed step wise with the clinical MAESTRO (MED-EL) software. Consequently, discrete steps during insertion were defined where the surgeon had to stop and the recording could be performed. Therewith insights were sought into the behavior of the OR amplitude in the following aspects: At first it was checked whether the OR amplitude rose during the insertion or not. The location of the electrode in the cochlea was calculated in order to determine whether the frequency generator has been passed by the recording contact. After the insertion the IC ECochG recording were repeated on different electrode contacts which should lie roughly at the position of the recording contact 1 during the discrete insertion steps in order to simulate the course of this insertion again afterward. Consequently, as the electrode is static, there is no new change in IC location and dynamic interaction with IC structures during those measurements. Hence, only the distance to the generator should affect the results here. This can be an important information to separate whether a drop in the OR amplitude during insertion occurred due to an acute, reversible event (e.g., temporal blockage of the basilar membrane) or rather having a long-term effect (e.g., due to a trauma to the cochlea). The long-term data at the 6M appointment was included in order to investigate the long-term stability. Furthermore, the data was also related to the individual preservation of the residual hearing as measured in the PTA.

Our six data sets were grouped for IED into two cases with short IED (≤ 20 mm), two cases with medium IED (≤ 24 mm) and two cases with long IED (≤ 28 mm) where both patients received a fully implanted Flex28 electrode. In the data sets with the short and the medium IED the amplitude of the OR was found to behave as expected with rising amplitude deeper in the cochlea. In one of these four cases (S04, IED 19.6 mm) the residual low frequency hearing was fully preserved in the long-term observation, and in the other three cases the low frequency hearing was partly preserved. In both cases with the longer IED the amplitude first rose and in the second half of the insertion progress or in the deeper lying half of the electrode carrier dropped. Here the residual low frequency hearing was partly preserved for both cases.

One theory about a late drop in the amplitude is that in these cases the electrode tip has crossed the generator frequency (Buechner et al., 2022; Haumann et al., 2019; Saoji et al., 2023). When investigating the position of the electrode in the cochlea and matching this with the presumed location of the generator frequency this theory can be substantiated in five of our six cases. In no case with short or medium IED the recording contact crossed the generators of 500 Hz which was the stimulation frequency in all our cases. In all of these cases, the amplitude rose when going deeper into the cochlea. In these cases the presumed main generators of the 500 Hz response were not crossed according to the estimations. Nevertheless, there are possible effects which are still not known. If there are preserved hair cells (not necessarily preserved hearing) at higher center frequency (CF) location, they will respond to the 500 Hz stimulus. So, some of the generators may very well have been crossed. In both cases with long IED the generator frequency was presumably crossed. In one of these cases (S06, IED 26.1 mm, final position of contact 1 at 278 Hz), the amplitude rose until the generator was presumably crossed and then dropped down. At the postinsertion measurement, there was again the pattern that the OR response was still present in the basal half of the cochlea. Here it may really be that only the generator was crossed, as in case of an acute cochlear trauma the amplitude should have been vanished at that point. Nevertheless, after six months the OR signal vanished. The residual hearing had deteriorated, but was still measurable. Thus, in this case there was maybe no acute trauma during electrode insertion, but the hearing as well as the hair cell responses deteriorated due to postoperative processes.

In the other case with long insertion (S01, IED 26.6 mm, final position of contact 1 at 128 Hz), however, the generator was presumably crossed, but the amplitude dropped before coming close to it. In this case also the postinsertion measurement showed deteriorated OR amplitudes and the hearing threshold after surgery at TSO had dropped. Nevertheless, in the long term, the hearing had recovered partly and also there were ORs measurable at the 6M appointment, so any potential event leading to the drop did not completely affect the residual hearing. However, it is noticeable that the residual hearing was found in the low-frequency range and the OR responses at electrode 9, according to our calculations at 5,153 Hz, thus being in the high-frequency range. Nevertheless, the described approach assumes the peak of response to come from the position of the Greenwood function. With loud stimuli, this assumption is not necessarily viable. Instead, the position of maximal response will vary greatly depending on the extent of generators left. Also, with stimuli of high intensities the position of the maximum stimulation of the basilar membrane is shifted toward basal, perhaps by an octave or more compared to the CF region (Honrubia & Ward, 1968). Our findings with subject S01 would be consistent with this interpretation.

Our data is complementary to the findings from Buechner et al. (2022) where the generator was presumably crossed in two patients. In one case, the OR response was stable and in the other case, the amplitude dropped and was accompanied by a large postoperative hearing loss. Our findings are also in line with Saoji et al. (2023) who stated based on the electrophysiological data that a comparison between recorded amplitudes during and after insertion can help to separate a trauma from a presumed crossing of the generators.

Another theory is that if the OR amplitude drops in the first half of the electrode insertion this most likely corresponds to transient disruptions, for example, a blockage of the basilar membrane, and a drop in the second half of the insertion could correspond to severe cochlear trauma which also leads to a deteriorated hearing (Koka et al., 2018; Sijgers et al., 2021). Our case with the drop before the presumed generator location was reached (S01) fits into this scheme, however, the assessment via a postinsertion measurement seems to be more adequate.

Some research groups postulate to include phase shift information into the interpretation of OR amplitude changes (Buechner et al., 2022; Giardina et al., 2019; Koka et al., 2018). Various theories have been put forward about the exact relationships, but it is assumed that a drop in OR amplitude with a phase shift could have more transient reasons, while a drop in OR amplitude without a phase shift could be associated with a cochlear trauma leading to residual hearing loss. At the time of measurement, the recordings during insertion via the electrode contacts using the clinical MAESTRO (MED-EL) software did not include an exact determination of the phase. Visual inspection of our data showed that in one of our cases with a drop in amplitude (S01, IED 26.6 mm) the phase changed during the drop and changed back afterward. In the other case (S06, IED 26.1 mm) the phase did not change during the insertion. Concerning the measured OR responses at the six month appointment, our findings are in line with these theories, concerning residual hearing not, as both patients had a comparable deterioration, but still measurable hearing after six months. Nevertheless, in the new research tool this analysis is included enabling us to deeper investigate this aspect in our next study.

With the repeated recording at the 6M appointment, the long-term stability of the results shall be investigated. In the time between the postinsertion measurement and the 6M appointment there were no more manipulations of the implant electrode performed, so different results should reflect only postoperative processes like inflammation, tissue grows, impedance changes, and so on. However, quantitative comparisons are difficult because the stimulation level may have been adjusted for the 6M measurement. The adjustments aimed for compensating changes in residual hearing, but nevertheless only qualitative comparisons should be made. In our four subjects with short and medium IED the shape of the OR amplitude curve over the frequencies looked similar, whereas in the two cases with long IED, the curves differed with mainly dropping down.

Another aspect is that the indications for the different IEDs are based on cochlear anatomy as well as residual hearing. In our four cases with short or medium IED, the pure tone audiogram showed good residual hearing in the low-frequency range and a steep slope in the high-frequency range, thus being ideal audiograms for electric-acoustic hearing. In the two cases with the long IED (fully inserted Flex28 electrode) the preoperative hearing curve was more or less pantonal and there was less residual hearing. It would also be possible that the increasing amplitude curve was related to the good residual hearing in the low-frequency range and the somewhat unclear curves with the long electrodes were related to the not-so-good residual hearing in the low-frequency range. Nevertheless, this remains to be investigated in a larger group of patients.

For the determination of the electrode location in the cochlea, it was decided to use the frequency and not the insertion angle. This was done to take into account the different sizes of the cochleae. For example when comparing the frequency of the insertion point “nine electrodes in” there was a large variety detected, ranging from 1.009 to 3.215 Hz. Furthermore, with this approach, the data can be better compared to the audiograms. What is noticeable also outside this analysis is that in some patients the amplitude hardly changes for a long time during insertion and then suddenly increases just before reaching the final position whereas in many patients the amplitude rises much earlier. However, when our four datasets with short and medium IED were examined more closely in this respect, the differences within this group could not be sufficiently explained from the audiogram. Especially in S02 with 17.3 mm IED the OR amplitude rose very early compared to the other subjects, but all four of them had similar breakpoint frequencies in the audiogram. In any case, it should be further investigated whether the model assumptions for the frequencies were correct or whether there are other aspects that could explain this.

One aspect that has to be regarded is that ECochG and PTA measure different entities, thus preserved ORs do not necessarily mean preserved hearing. ECochG is measured in or close to the cochlea, reflecting the state of the hair cells and some neural structures depending on the recorded component of the potential, but with PTA the entire process of hearing is measured. There could for example be effects of CM responses due to surviving hair cells, but deterioration of neural structures could result in the patient not hearing the sound (Campbell et al., 2015; Fitzpatrick et al., 2014; Haumann et al., 2019). Another aspect is the different time course. PTA is conducted before surgery and 1–3 days after surgery, whereas the ECochG recordings are conducted intraoperatively during and after CI electrode insertion. Thus, in case of a good OR at the end of the surgery, but deteriorated PTA afterwards it can hardly be separated whether the postinsertion recording yielded a false positive response or the residual hearing deteriorated due to postoperative processes. Nevertheless, studies investigating the relation between postoperatively recorded ECochG thresholds and PTA at the same appointment show high correlation (Buechner et al., 2022; Haumann et al., 2019; Krüger et al., 2020). Thus, it can be assumed with some certainty that in case of a good OR response at the end of the CI electrode insertion the residual hearing would still be present at this moment and a possible deterioration of residual hearing could probably attributed to postoperative processes.

## Conclusion

In this study, IC ECochG recordings were retrospectively analyzed where the integrated hard- and software solution of a CI company was used. In all subjects, ORs could be measured throughout the cochlea. In the four cases with a typical audiometric threshold for EAS, the OR amplitude rose during the insertion, whereas the behavior of the OR amplitude was not that clear for the two cases with pantonal hearing loss. In one case the generator appears to have been crossed, as the postinsertion recording on different electrode contacts showed the same amplitude pattern as the recording during insertion, and in one case there may have been trauma, as the drop occurred before the generator was presumably reached and the post insertion recording showed deteriorated amplitudes compared to the recording during insertion.

In our approach, the behavior of the OR amplitude during insertion was evaluated by repeating the recording after insertion on different electrode contacts throughout the cochlea, therewith simulating the course of this insertion again afterward in static conditions. This approach seems to be useful to separate whether a drop in the OR amplitude during insertion was caused by temporal occurrences like a blockage of the basilar membrane or by an incident that would lead to a deterioration of the postoperative hearing.

Thus, the combination of IC ECochG with the determination of the electrode position in the cochlea offers promising possibilities for cochlear monitoring during CI surgery. Based on our data, we recommend matching the position of the electrode in the cochlea with the generator frequency in the future in order to rule out crossing the generator frequency as the cause of the amplitude drop.

## Supplemental Material

sj-docx-1-tia-10.1177_23312165241248973 - Supplemental material for Intracochlear Recording of Electrocochleography During and After Cochlear Implant Insertion Dependent on the Location in the Cochlea Supplemental material, sj-docx-1-tia-10.1177_23312165241248973 for Intracochlear Recording of Electrocochleography During and After Cochlear Implant Insertion Dependent on the Location in the Cochlea by Sabine Haumann, Max E. Timm, Andreas B?, Thomas Lenarz and Rolf B. Salcher in Trends in Hearing
