# Supplementary material for: Intracochlear Recording of Electrocochleography During and After Cochlear Implant Insertion Dependent on the Location in the Cochlea
Source: Trends Hear. 2024 May 8;28:23312165241248973. doi: 10.1177/23312165241248973 (PMC11080744; doi:10.1177/23312165241248973)
Supplement: sj-docx-1-tia-10.1177_23312165241248973 - Supplemental material for Intracochlear Recording of Electrocochleography During and After Cochlear Implant Insertion Dependent on the Location in the Cochlea [file sj-docx-1-tia-10.1177_23312165241248973.docx]

# Intracochlear recording of Electrocochleography during and after cochlear implant insertion dependent on the location in the cochlea

Sabine Haumann, Max E. Timm, Andreas Büchner, Thomas Lenarz, Rolf B. Salcher

**2024**

## Supplementary Material

# Methods and Materials

**Noise floor estimation**

All objective measurements suffer from a certain noise level, which depends on several factors like measurement setup, surroundings, state of the patients, number of the recorded averages per trial, filter settings and others. In this investigation intracochlear (IC) recordings using CI hard- and software from the company MED-EL (Innsbruck, Austria) were done and the Nicolet Viking EDX system (Natus Medical Incorporated, Pleasanton, CA, USA) was used for stimulating. Intraoperative recordings were done in a noisy operating theatre and 50 averages per trial were used. Postoperative recordings during the follow up were done either in an electrically shielded measurement cabin or in a quiet room of our German Hearing Center which is well separated from noisy medical equipment in the main building of our clinic.

For estimating noise floors for our IC setup with MED-EL implants, the data from a study described in an earlier work with 10 patients^[[1]](#footnote-1)^ was used. Here, the recordings were done at different appointments and with three stimulating frequencies (250 Hz, 500 Hz, 1000 Hz). In that manuscript, the noise floor estimation was described and given for one example patient. Within each recording session, a trial with 0 dB nHL stimulation was performed and processed in the same way than the trials with higher stimulation levels which was also applied in the data presented here. Within this processing a Fast Fourier Transform (FFT) was performed. In the cited manuscript, the amplitudes of all bins from the FFT were averaged that lay within the filter limits, but for the current work the procedure was changed. Now, only data from the 0 dB recordings with 500 Hz stimulation frequency were used, and the amplitudes were averaged over the ten subjects for each frequency bin of the FFT. This was calculated separately for the intraoperative recordings with 50 averages and a follow up recording with 100 averages. To decide whether a stimulus response is present or absent, it has to be investigated whether the recorded signal exceeds the noise level. For this, a 99% confidence interval was calculated as µ ± 2.576 * σ and a response was accepted when the amplitude exceeded the upper limit of this confidence interval. The estimated noise floor for intraoperative and follow up recordings is given in **Figure 1**.


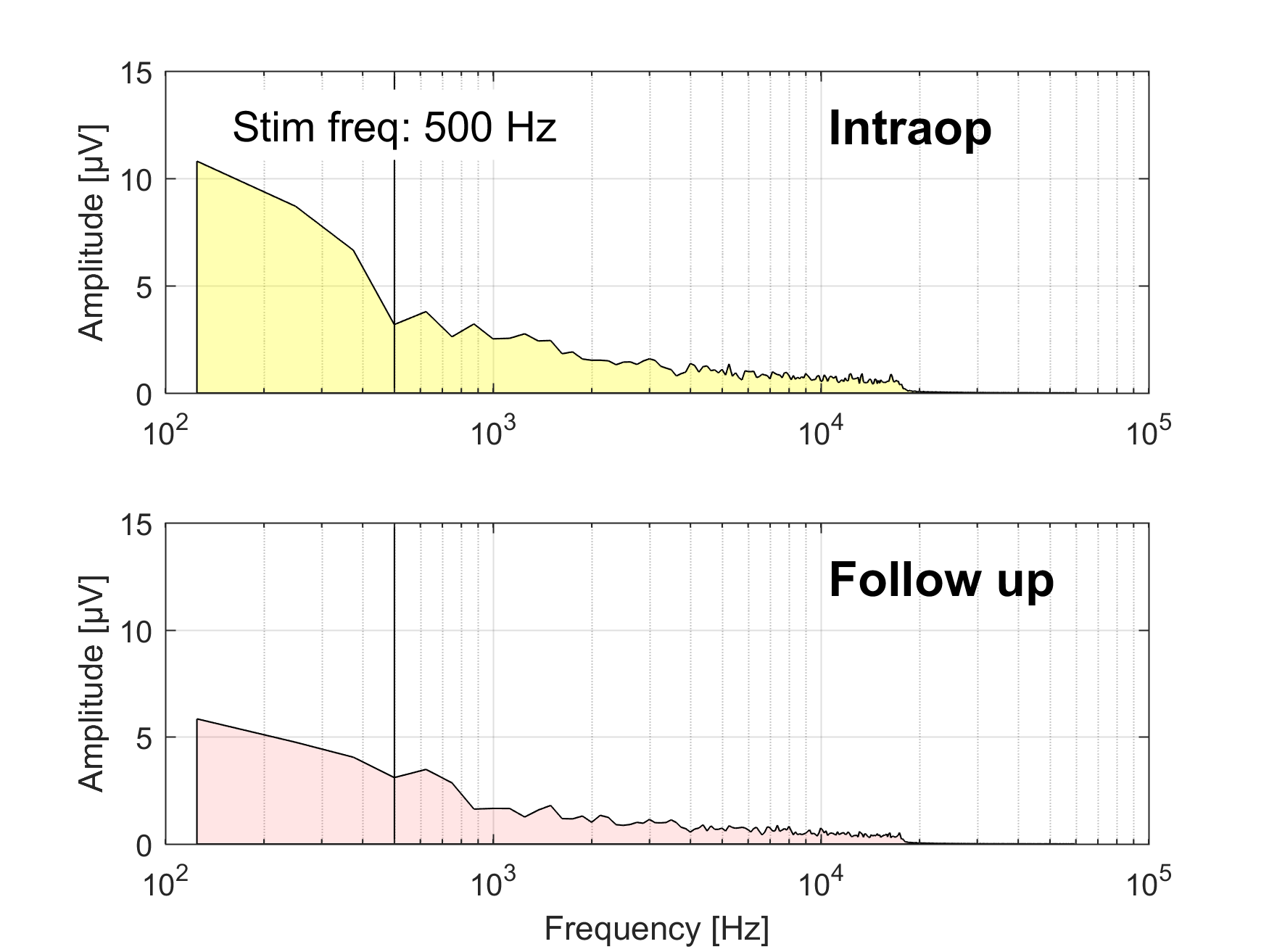


**Figure 1:** Noise floor estimations for n=10 subjects. The data is from our predecessor study (Haumann et al, 2019). Noise floor calculation is separated for intraoperative recordings (n=50 traces per average) given in the upper panel and follow up recordings (n=100 traces per average) given in the lower panel. The vertical black line represents the stimulation frequency (500 Hz).

# Results

**Data of all individual subjects**

In the following, the data for all individual subjects is given. The structure of the graphics is always the same, which is why the structure is described in detail in the captions of the data from the first patient and from the second patient onwards only individual settings are highlighted.

*Subject 1*

S01 was a female subject, 71.9 years old at the time of the surgery, receiving a Flex28 electrode with 26.6 mm Inserted Electrode Depth (IED) on the right side. The intraoperative data during insertion is given in **Figure 2**, the intraoperative data after insertion in **Figure 3** and the follow up data as measured at the 6 months appointment in **Figure 4**.


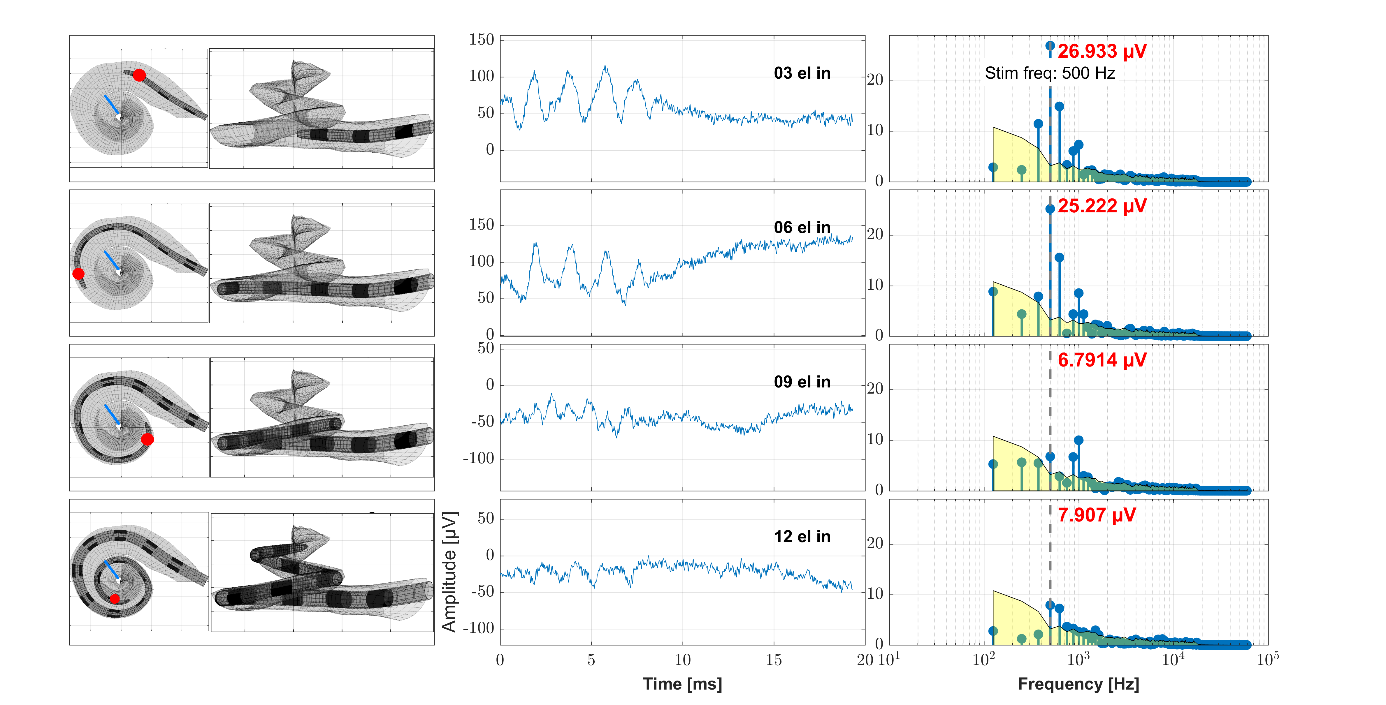


**Figure 2:** S01: Intraoperatively recorded data during CI electrode insertion. The stimulation was done using 500 Hz tone bursts with 99 dB nHL. The recordings took place at discrete electrode insertion steps using the most apical contact C1. On the left panels these steps are visualized by means of the assumed electrode insertion depth as calculated from the preoperative clinical imaging data with the recording contact being highlighted^[[2]](#footnote-2)^. The blue line represents the presumed location in the cochlea of the 500 Hz characteristic frequency region. On the middle panels the time signal of the ongoing response (OR) is shown and on the right panels the frequency analysis by FFT. The grey vertical dashed line represents the stimulus frequency (500 Hz), the red number at each FFT plot states the amplitude at the according frequency bin and the yellow area visualizes the noise floor. From top to down the ongoing CI electrode insertion is shown.


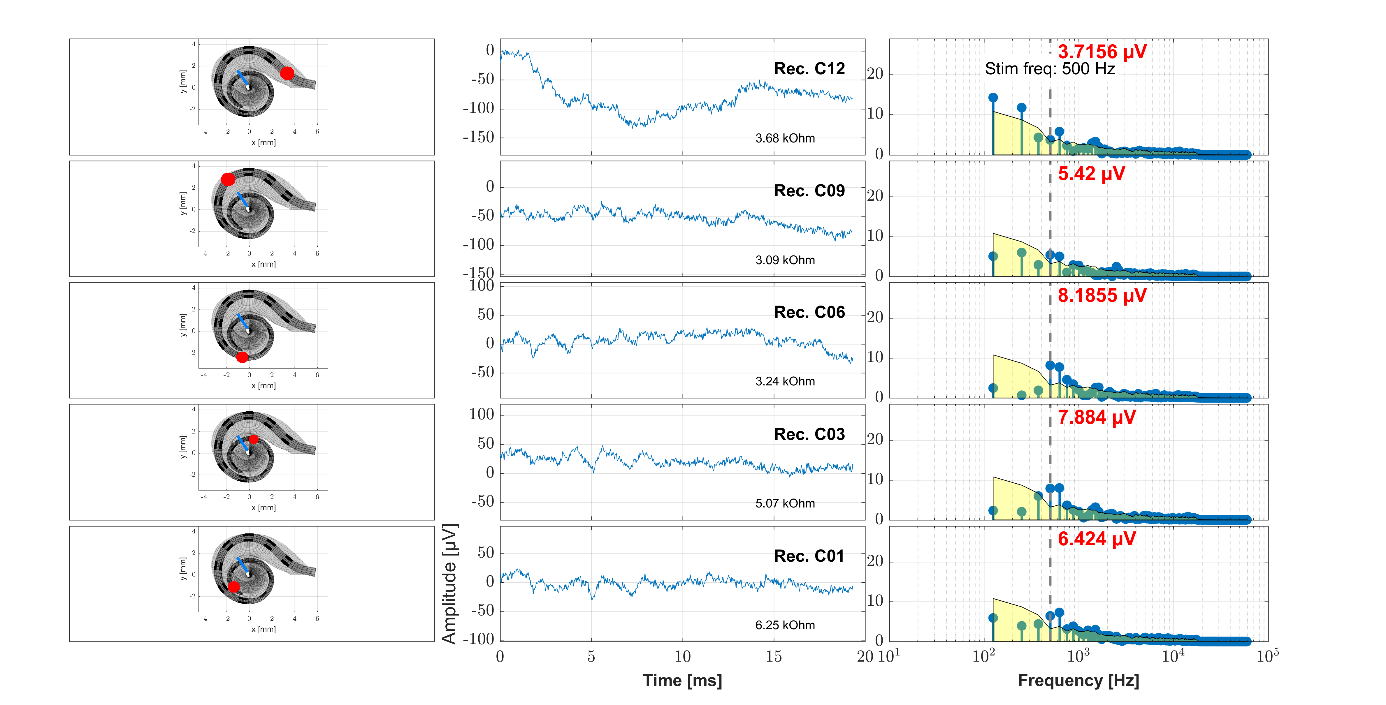


**Figure 3:** S01: Intraoperatively recorded data directly after CI electrode insertion where the recording was performed at different recording contacts. The stimulation was done using 500 Hz tone bursts with 99 dB nHL. On the left panels the position of the electrode in the cochlea as measured from the postoperative clinical imaging data is shown with the current recording contact being highlighted. The blue line represents the presumed location in the cochlea for the 500 Hz characteristic frequency region. On the middle panels the time signal of the ongoing response (OR) is shown and on the right panels the frequency analysis by FFT. The grey vertical dashed line represents the stimulus frequency (500 Hz), the red number at each FFT plot states the amplitude at the according frequency bin and the yellow area visualizes the noise floor. From top to down the data obtained at different recording contacts is shown. Also the impedance of each recording contact is given.


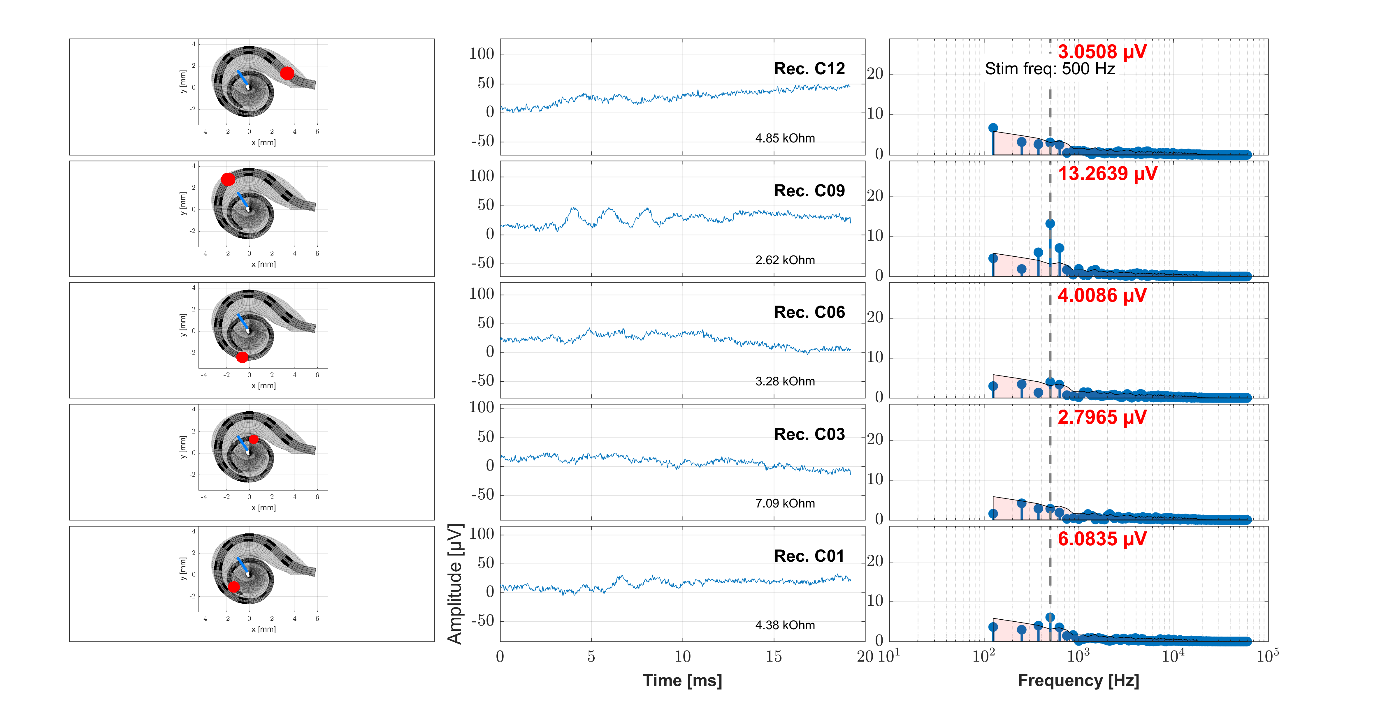


**Figure 4:** S01: Data recorded at the follow up appointment after six months of CI use where the recording was performed at different recording contacts. The stimulation was done using 500 Hz tone bursts with 95 dB nHL. On the left panels the position of the electrode in the cochlea as measured from the postoperative clinical imaging data is shown with the current recording contact being highlighted. The blue line represents the presumed location in the cochlea for the 500 Hz characteristic frequency region. On the middle panels the time signal of the ongoing response (OR) is shown and on the right panels the frequency analysis by FFT. The grey vertical dashed line represents the stimulus frequency (500 Hz), the red number at each FFT plot states the amplitude at the according frequency bin and the light red area visualizes the noise floor. From top to down the data obtained at different recording contacts is shown. Also the impedance of each recording contact is given.

*Subject 2*

S02 was a female subject, 42.7 years old at the time of the surgery, receiving a Flex20 electrode with 17.3 mm IED on the right side. This subject was the example patient in the main manuscript, so two of these figures are already presented there, but for clarity they are added here too. The intraoperative data during insertion is shown in **Figure 5**, the intraoperative data after insertion is shown in **Figure 6** and the postoperative data is shown in **Figure 7**.


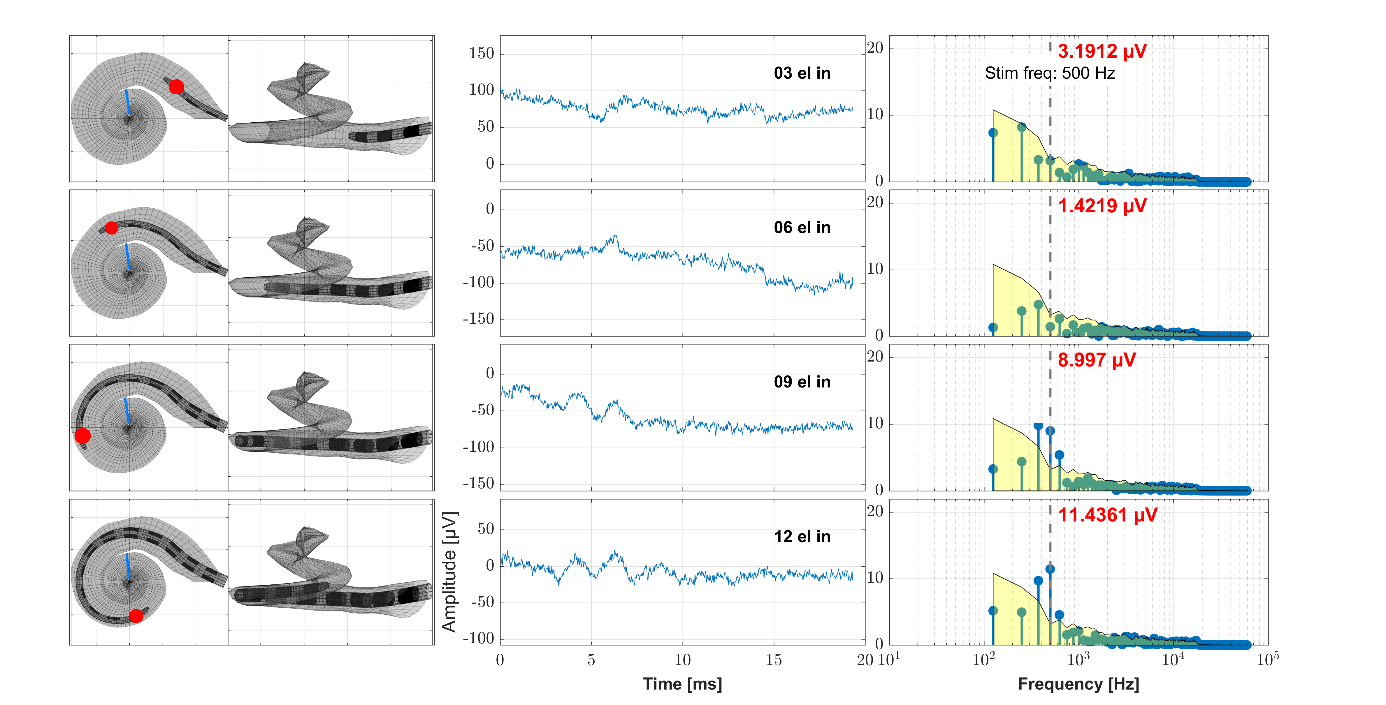


**Figure 5:** S02: intraop during insertion, 500 Hz, 70 dB nHL


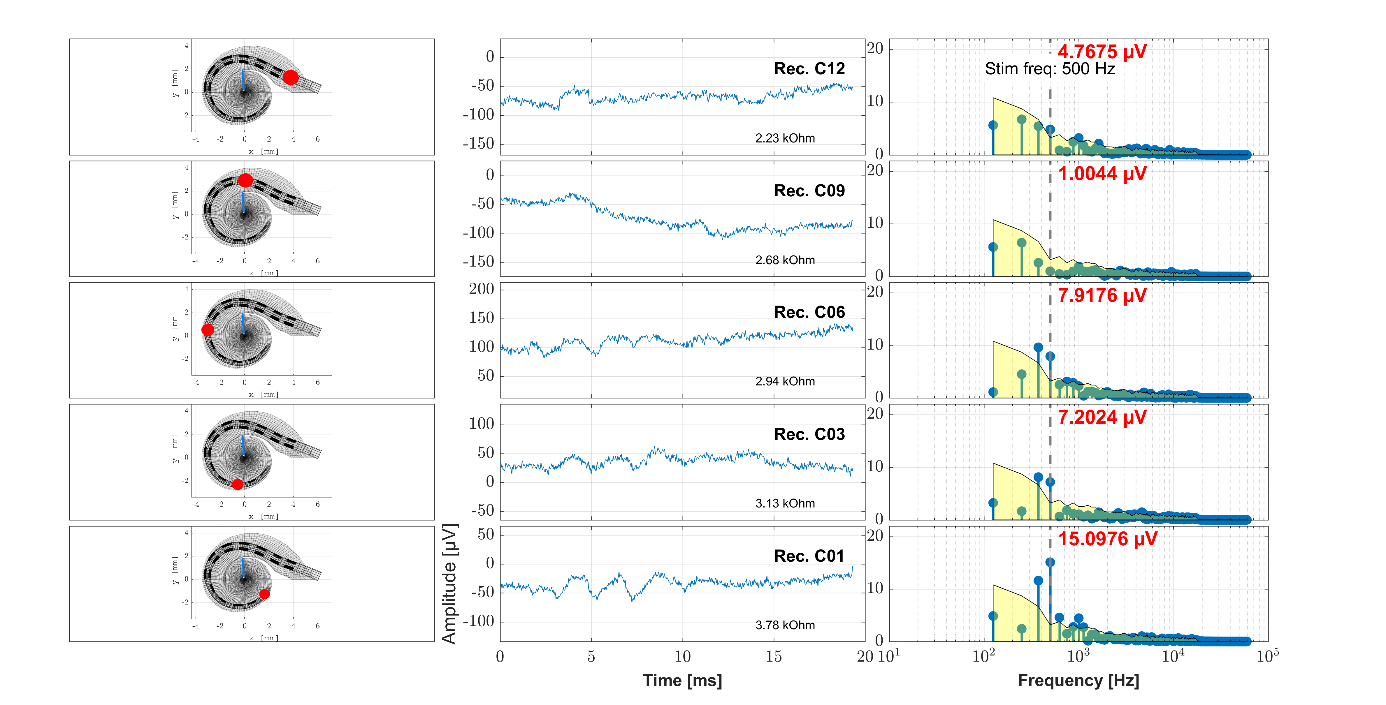


**Figure 6:** S02: intraop after insertion, 500 Hz, 70 dB nHL


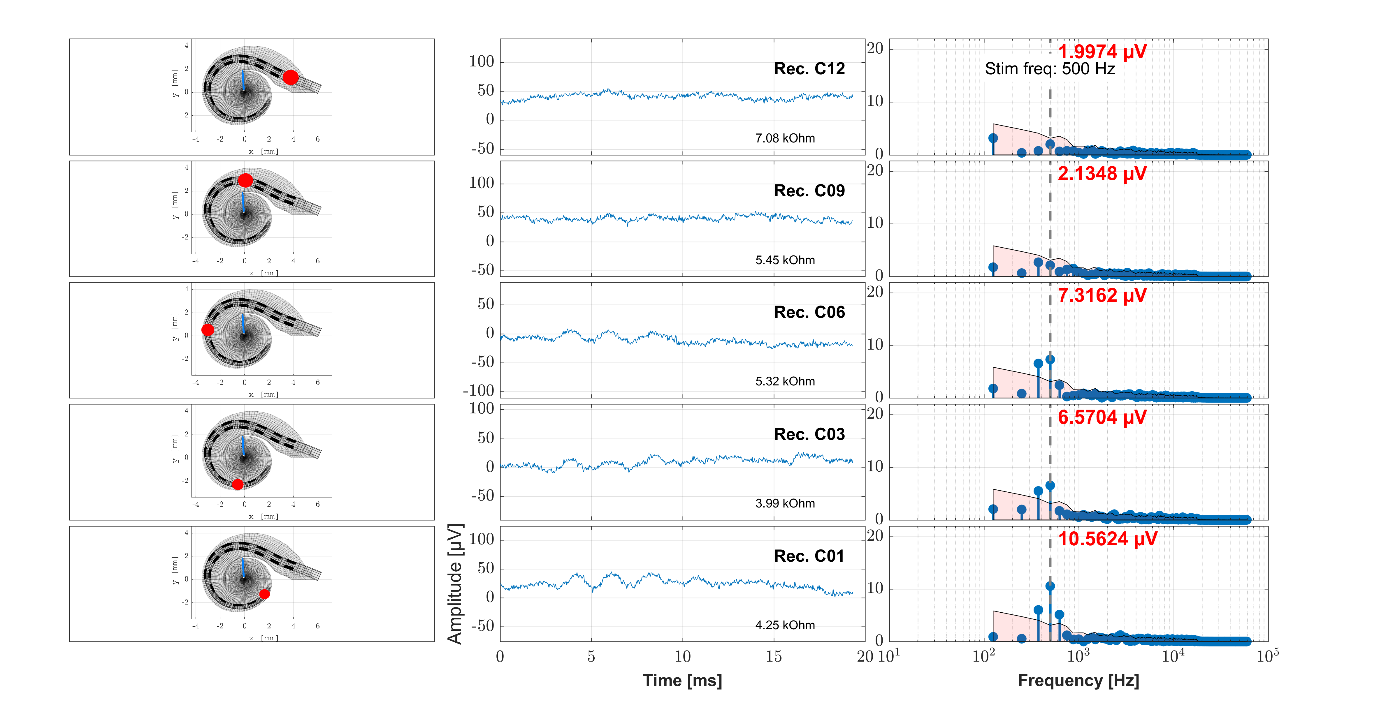


**Figure 7:** S02: postop after six months, 500 Hz, 70 dB nHL

*Subject 3*

S03 was a female subject, 43.3 years old at the time of the surgery, receiving a Flex28 electrode with 22.1 mm IED on the right side. The intraoperative data during insertion is shown in **Figure 8**, the intraoperative data after insertion is shown in **Figure 9** and the postoperative data is shown in **Figure 10**.


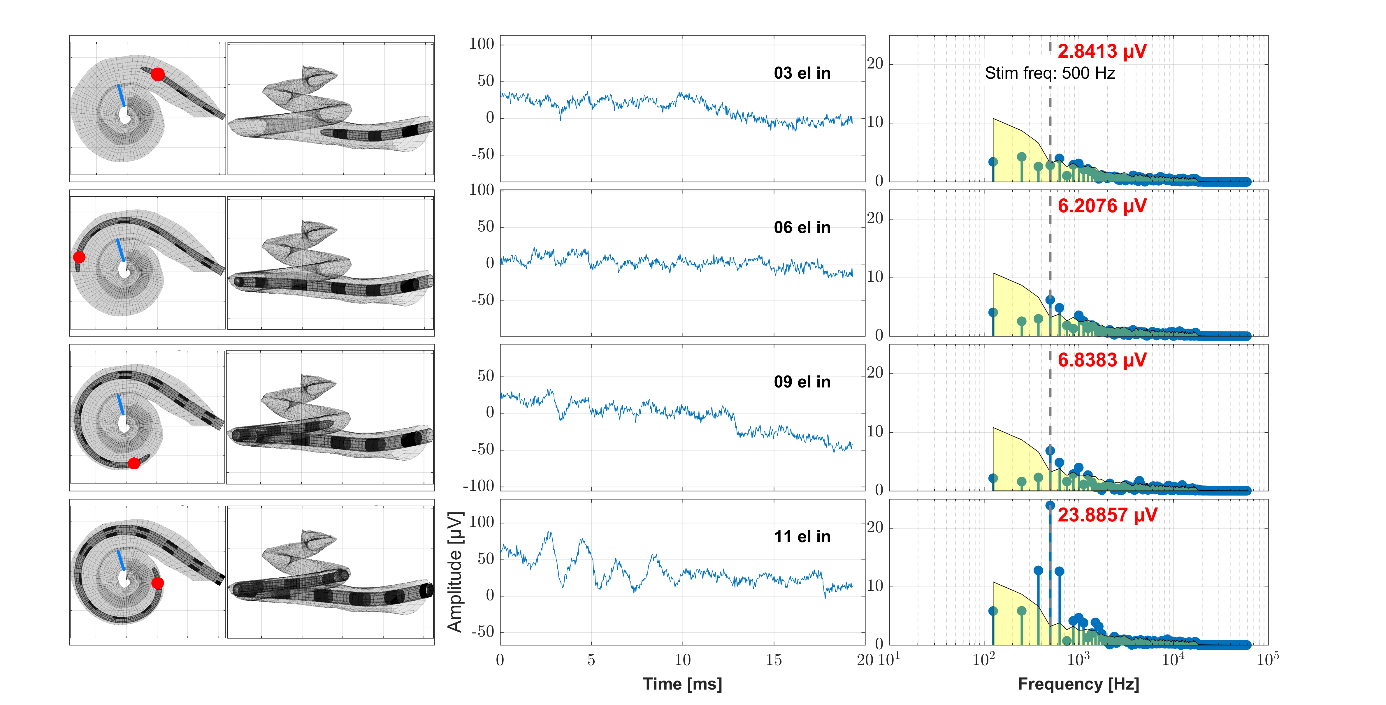


**Figure 8:** S03: intraop during insertion, 500 Hz, 90 dB nHL


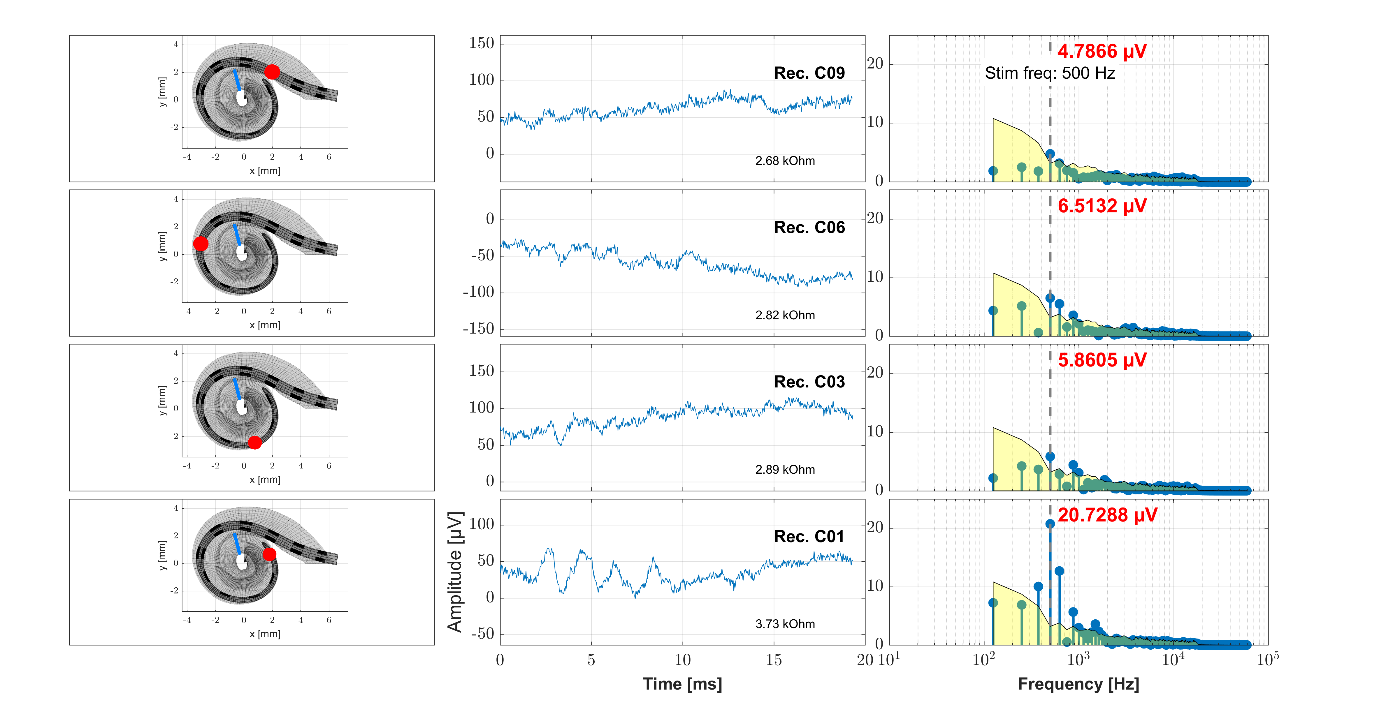


**Figure 9:** S03: intraop after insertion, 500 Hz, 90 dB nHL


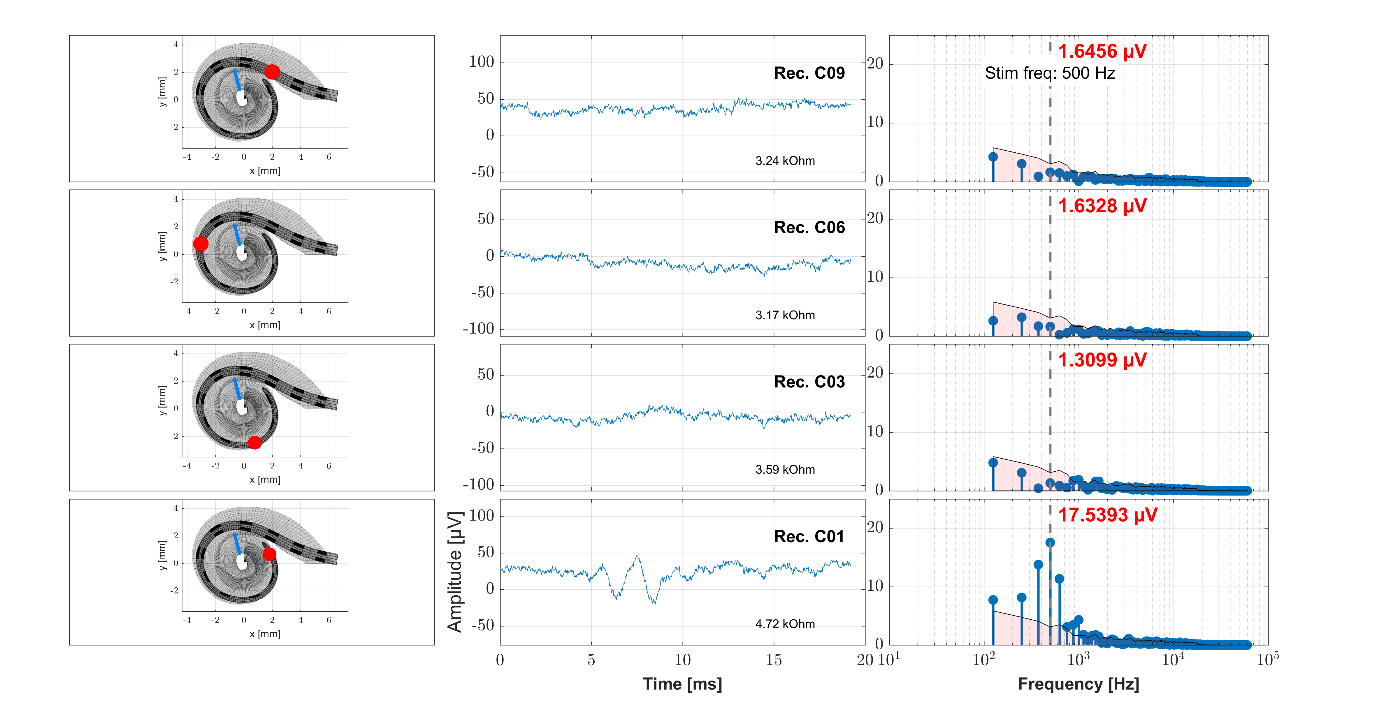


**Figure 10:** S03: postop after six months, 500 Hz, 82 dB nHL

*Subject 4*

S04 was a female subject, 75.3 years old at the time of the surgery, receiving a Flex28 electrode with 19.6 mm IED on the left side. The intraoperative data during insertion is shown in **Figure 11**, the intraoperative data after insertion is shown in **Figure 12** and the postoperative data is shown in **Figure 13**.


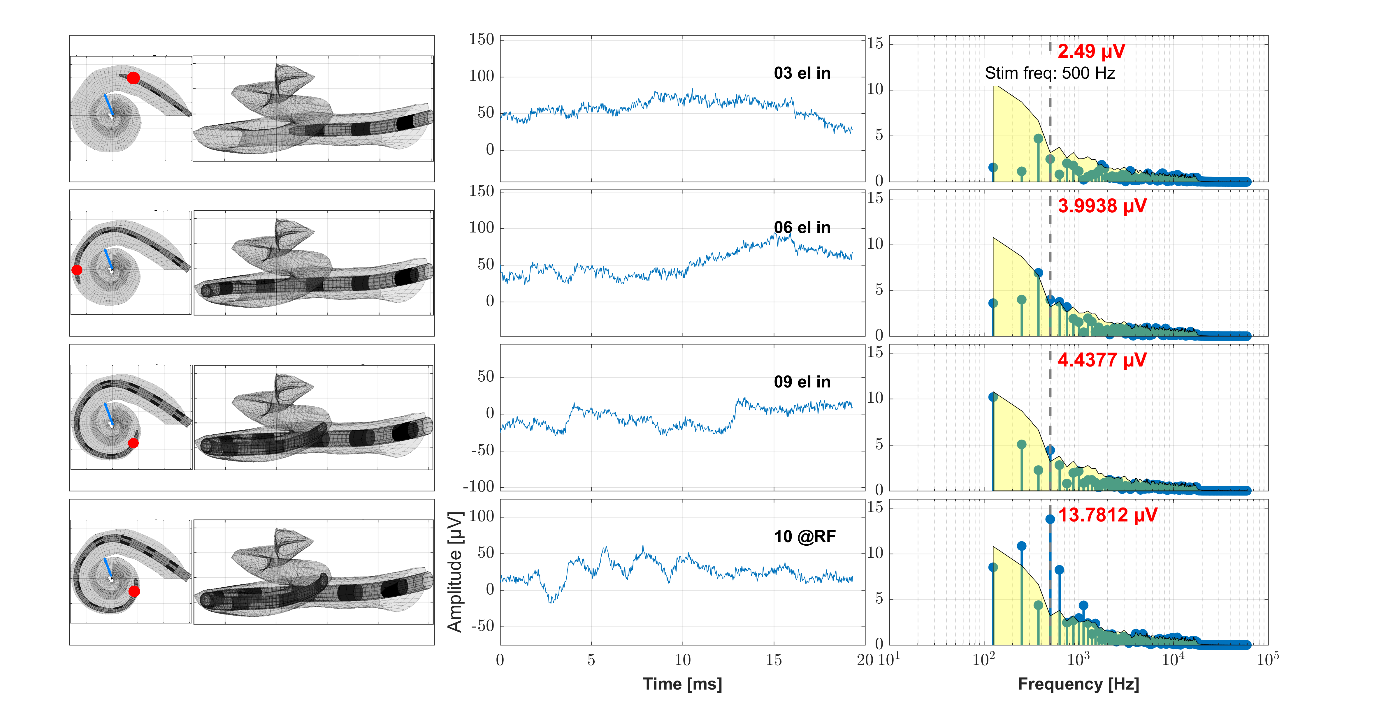


**Figure 11:** S04: intraop during insertion, 500 Hz, 90 dB nHL


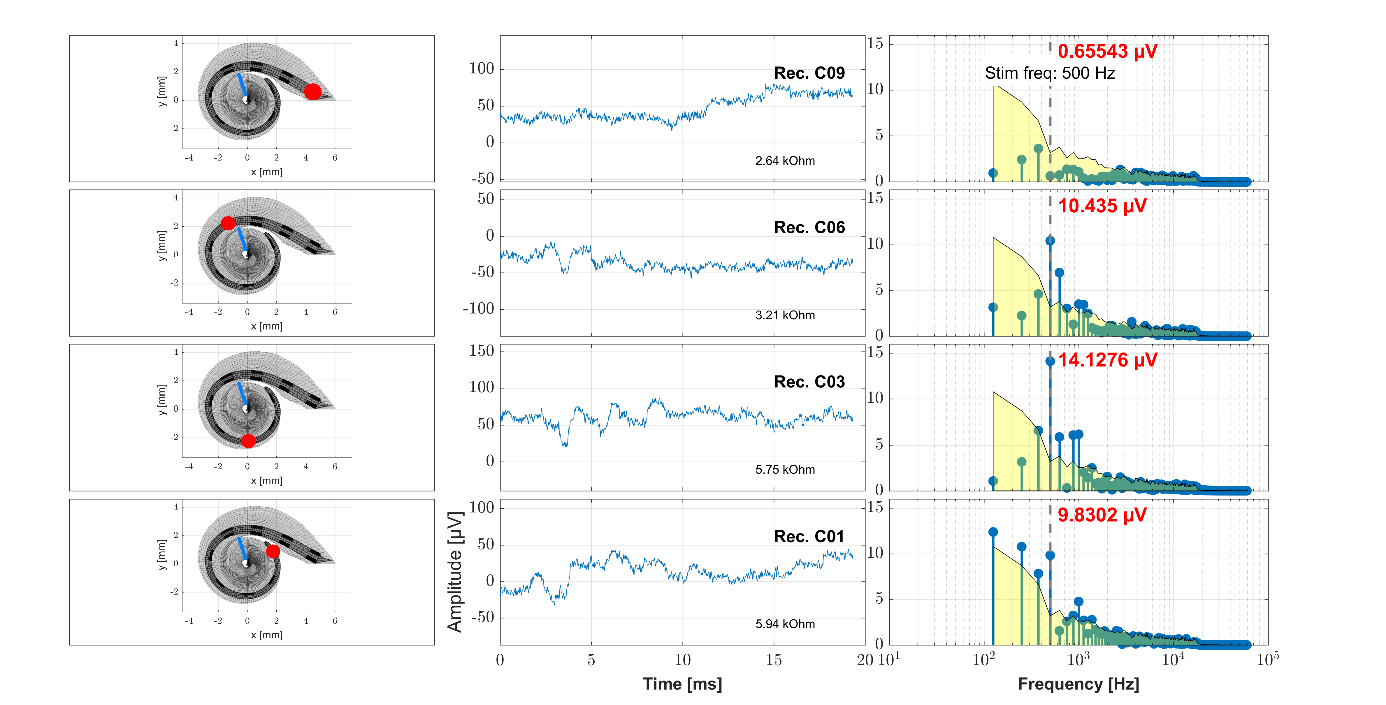


**Figure 12:** S04: intraop after insertion, 500 Hz, 90 dB nHL


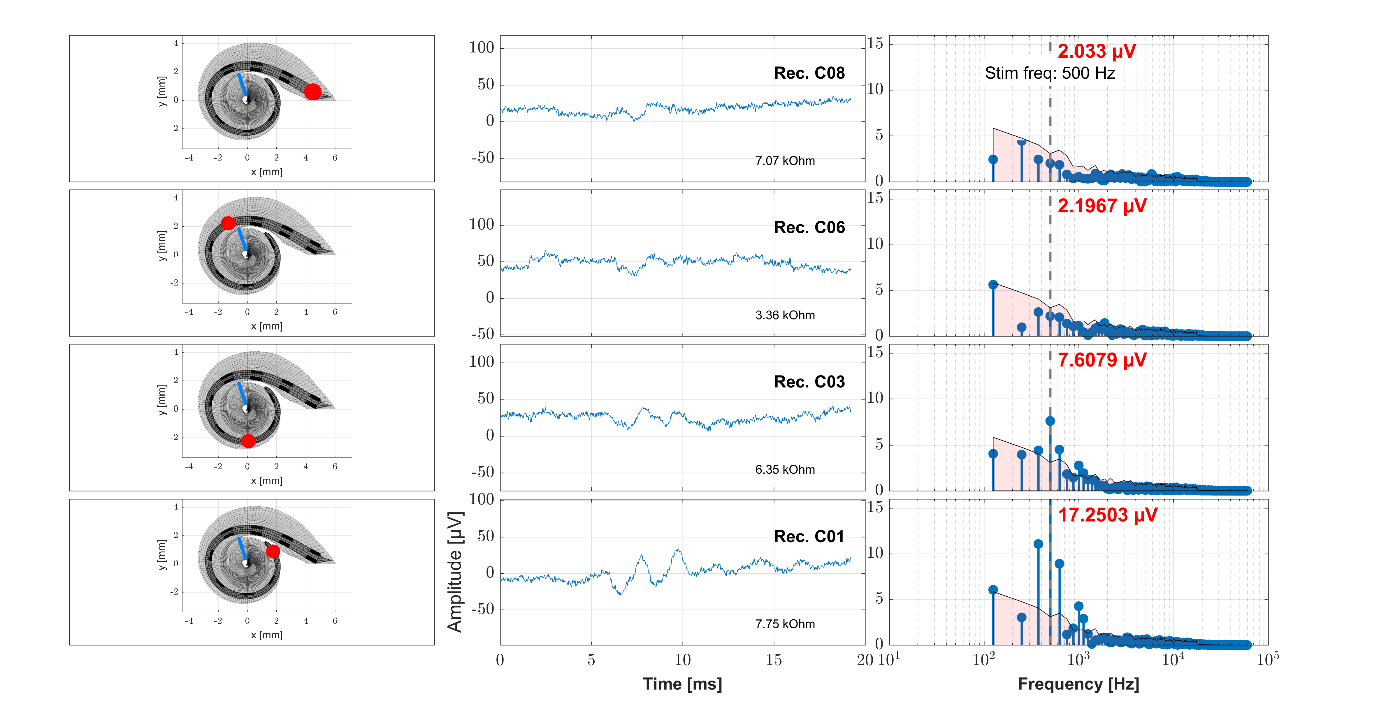


**Figure 13:** S04: postop after six months, 500 Hz, 80 dB nHL

*Subject 5*

S05 was a female subject, 56.7 years old at the time of the surgery, receiving a Flex28 electrode with 21.5 mm IED on the left side. The intraoperative data during insertion is shown in **Figure 14**, the intraoperative data after insertion is shown in **Figure 15** and the postoperative data is shown in **Figure 16**.


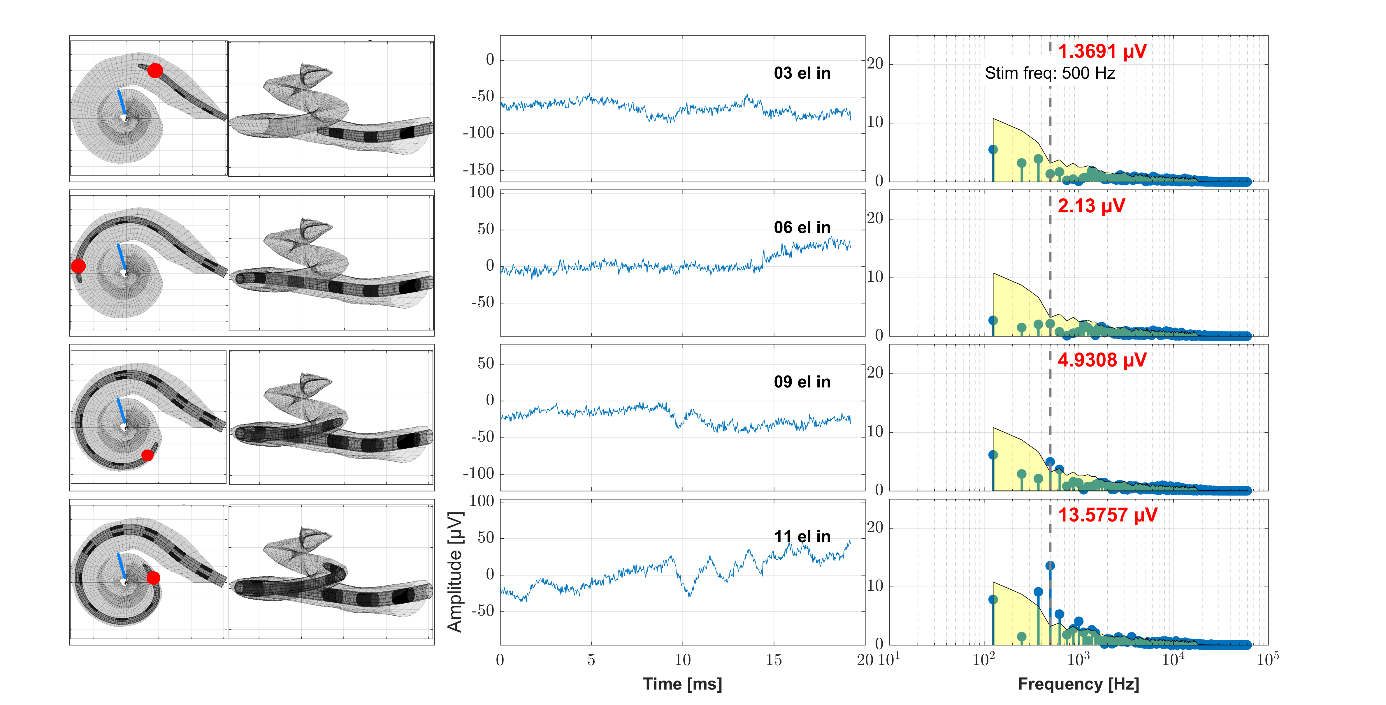


**Figure 14:** S05: intraop during insertion, 500 Hz, 70 dB nHL


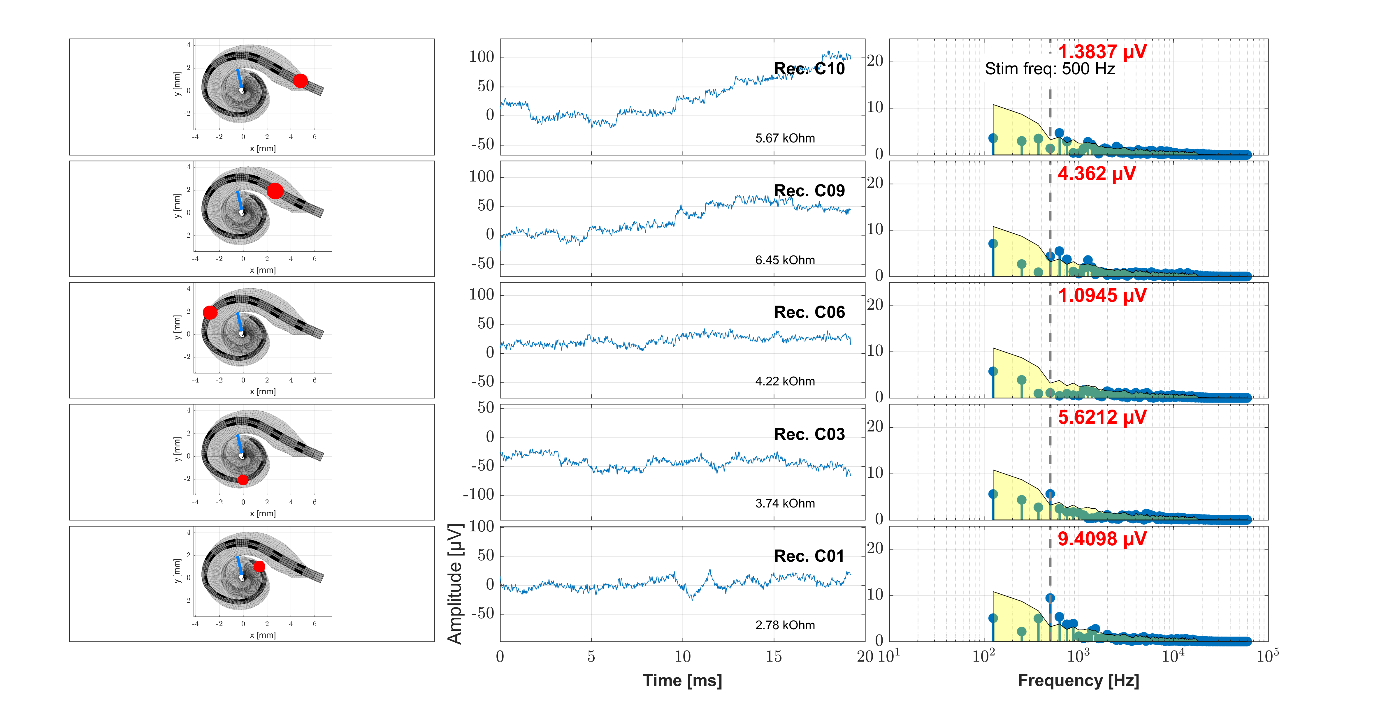


**Figure 15:** S05: intraop after insertion, 500 Hz, 70 dB nHL


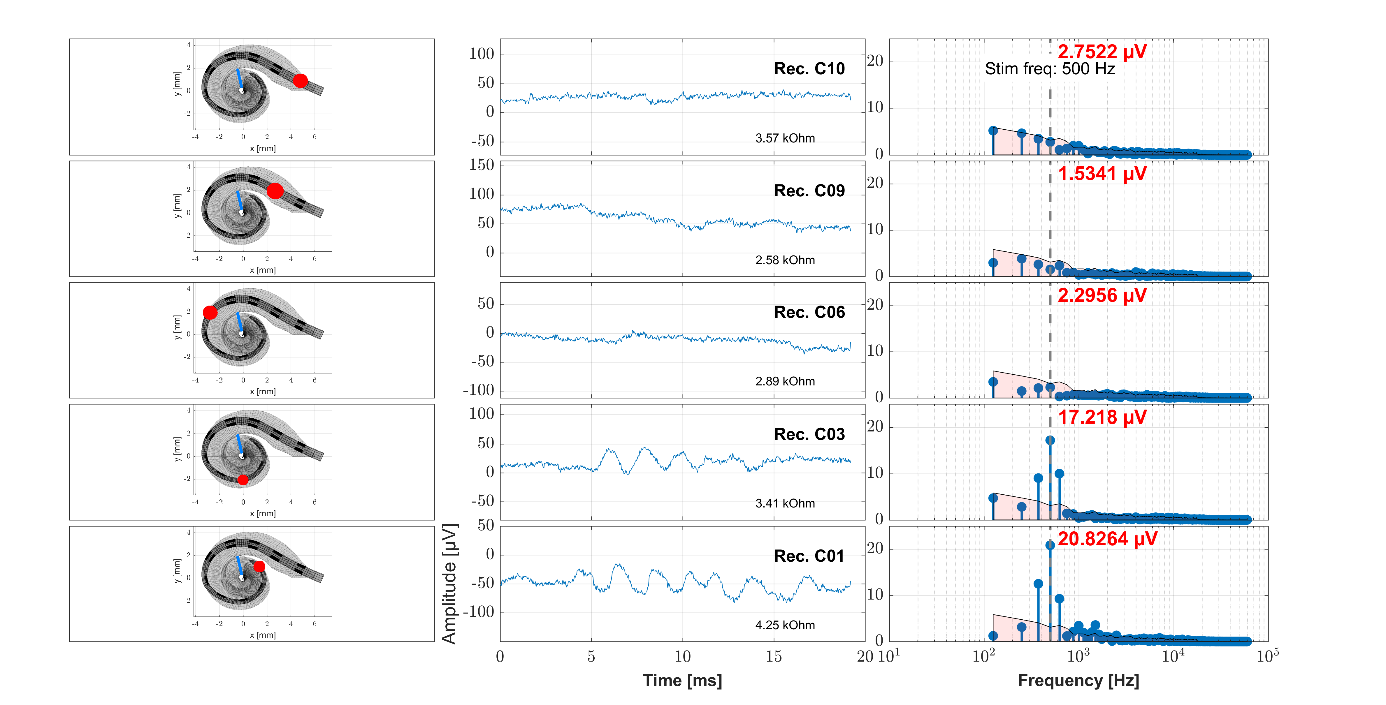


**Figure 16:** S05: postop after six months, 500 Hz, 80 dB nHL

*Subject 6*

S06 was a male subject, 71.9 years old at the time of the surgery, receiving a Flex28 electrode with 26.6 mm IED on the right side. The intraoperative data during insertion is shown in **Figure 17**, the intraoperative data after insertion is shown in **Figure 18** and the postoperative data is shown in **Figure 19**.


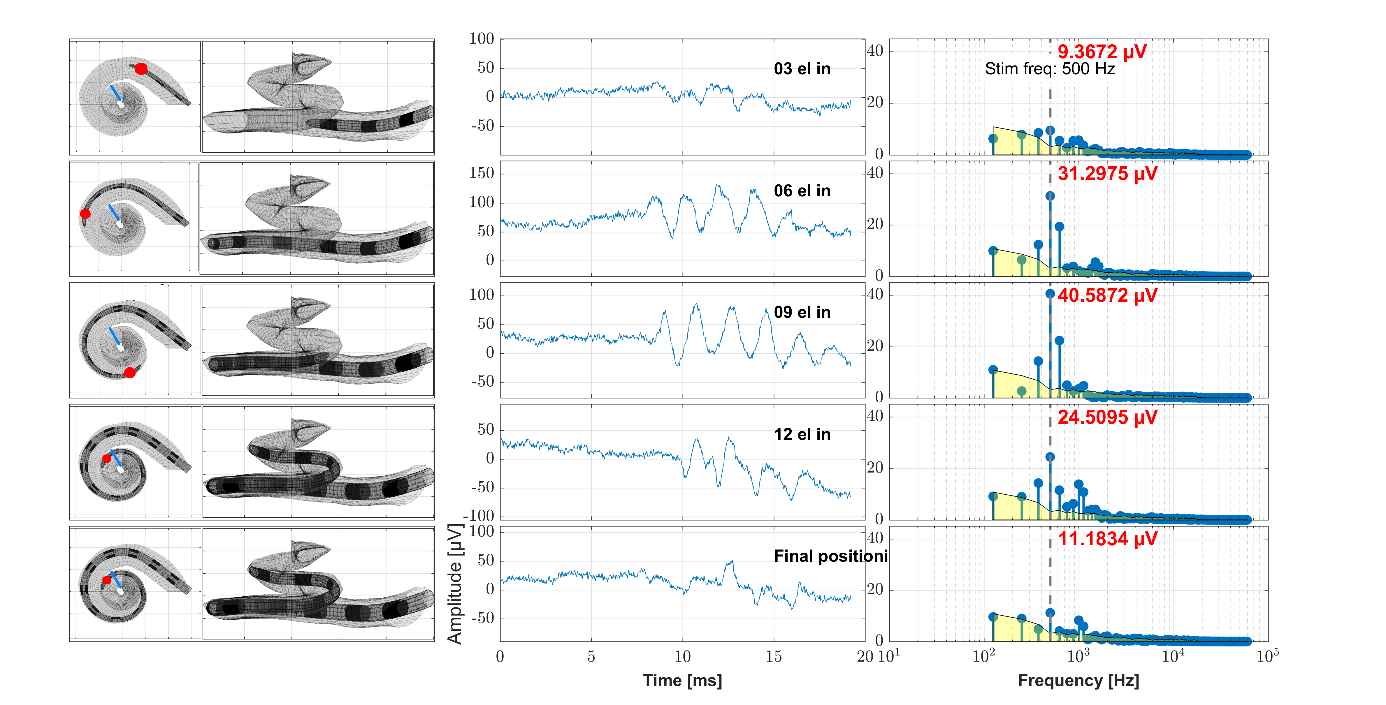


**Figure 17**: S06: intraop during insertion, 500 Hz, 90 dB nHL


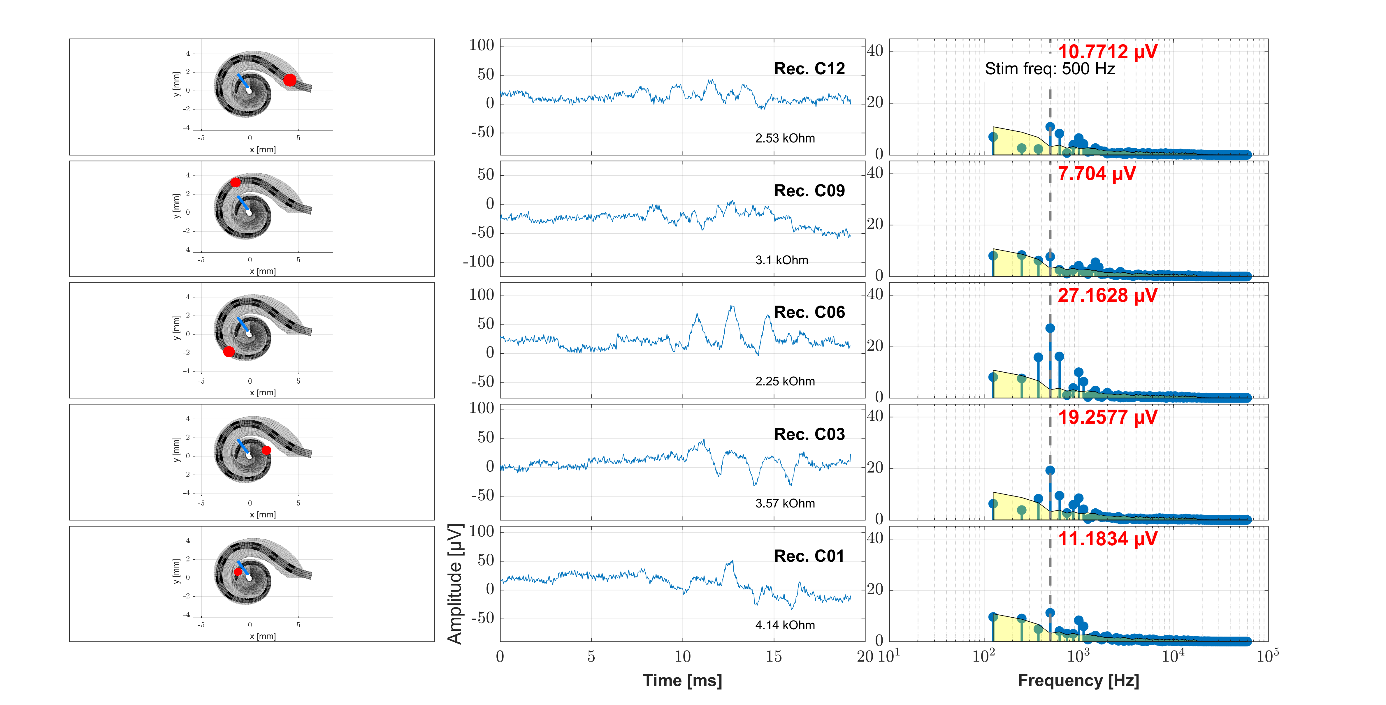


**Figure 18:** S06: intraop after insertion, 500 Hz, 90 dB nHL


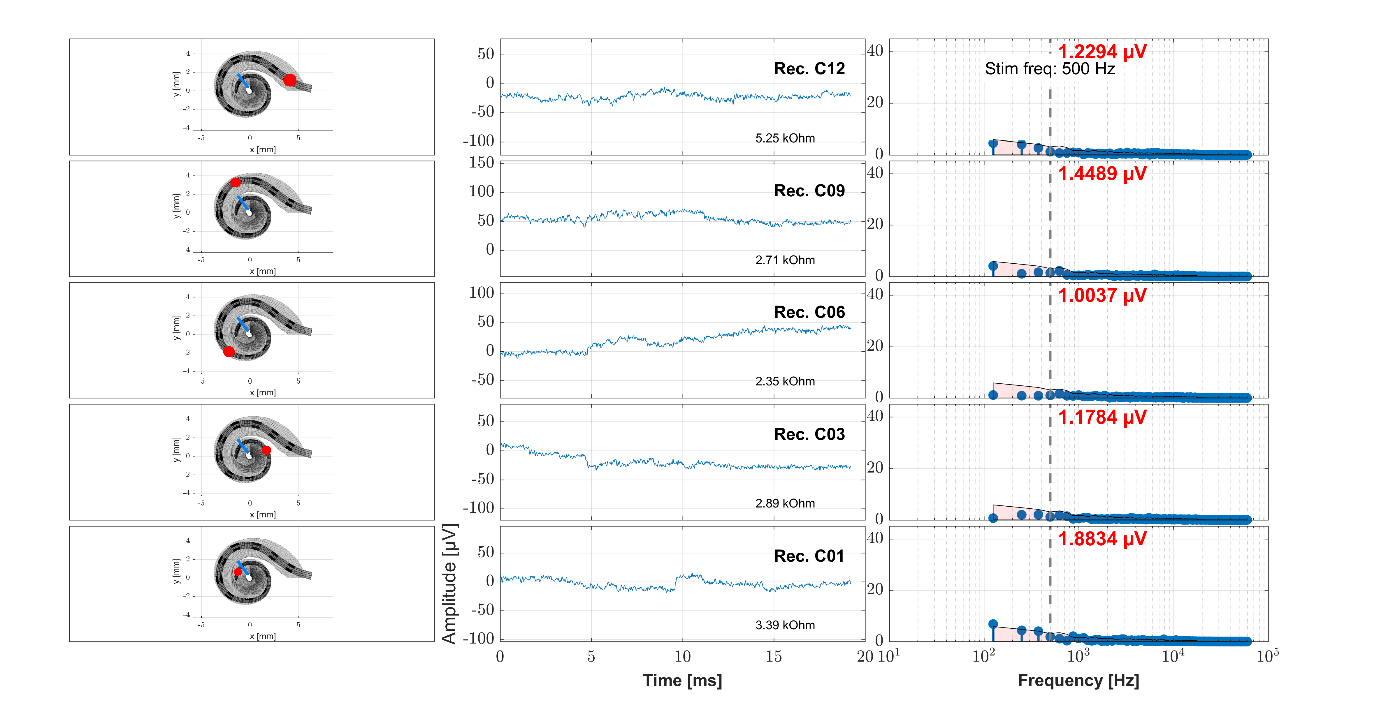


**Figure 19:** S06: postop after six months, 500 Hz, 95 dB nHL

1. S. Haumann, M. Imsiecke, G. Bauernfeind, A. Büchner, V. Helmstaedter, T. Lenarz, R.B. Salcher (2019): Monitoring of the Inner Ear Function During and After Cochlear Implant Insertion Using Electrocochleography. Trends in Hearing 23:2331216519833567. DOI: 10.1177/2331216519833567 [↑](#footnote-ref-1)
2. Schurzig, D., Repp, F., Timm, M. E., Batsoulis, C., Lenarz, T., & Kral, A. (2023). Virtual Cochlear Implantation for Personalized Rehabilitation of Profound Hearing Loss. *Hearing Research, 429*, 108687. [↑](#footnote-ref-2)
